# DNA Repair Molecular Beacon assay: a platform for real-time functional analysis of cellular DNA repair capacity

**DOI:** 10.18632/oncotarget.25859

**Published:** 2018-08-03

**Authors:** Jianfeng Li, David Svilar, Steven McClellan, Jung-Hyun Kim, Eun-Young Erin Ahn, Conchita Vens, David M. Wilson, Robert W. Sobol

**Affiliations:** ^1^ University of South Alabama Mitchell Cancer Institute, Mobile, AL, USA; ^2^ Department of Pharmacology & Chemical Biology, University of Pittsburgh School of Medicine, Pittsburgh, PA, USA; ^3^ University of Pittsburgh Cancer Institute, Hillman Cancer Center, Pittsburgh, PA, USA; ^4^ The Netherlands Cancer Institute, Division of Cell Biology, Amsterdam, The Netherlands; ^5^ Laboratory of Molecular Gerontology, National Institute on Aging, IRP, NIH Baltimore, MD, USA

**Keywords:** base excision repair, molecular beacon assay, microbead-conjugated molecular beacon assay, DNA glycosylase, APE1

## Abstract

Numerous studies have shown that select DNA repair enzyme activities impact response and/or toxicity of genotoxins, suggesting a requirement for enzyme functional analyses to bolster precision medicine or prevention. To address this need, we developed a DNA Repair Molecular Beacon (DRMB) platform that rapidly measures DNA repair enzyme activity in real-time. The DRMB assay is applicable for discovery of DNA repair enzyme inhibitors, for the quantification of enzyme rates and is sufficiently sensitive to differentiate cellular enzymatic activity that stems from variation in expression or effects of amino acid substitutions. We show activity measures of several different base excision repair (BER) enzymes, including proteins with tumor-identified point mutations, revealing lesion-, lesion-context- and cell-type-specific repair dependence; suggesting application for DNA repair capacity analysis of tumors. DRMB measurements using lysates from isogenic control and APE1-deficient human cells suggests the major mechanism of base lesion removal by most DNA glycosylases may be mono-functional base hydrolysis. In addition, development of a microbead-conjugated DRMB assay amenable to flow cytometric analysis further advances its application. Our studies establish an analytical platform capable of evaluating the enzyme activity of select DNA repair proteins in an effort to design and guide inhibitor development and precision cancer therapy options.

## INTRODUCTION

Advanced technologies for characterizing human populations at the molecular level have set the stage for personalized therapeutic strategies [1–4]. These may also provide an opportunity for personalized prevention, as we have described [5]. Preclinical and clinical studies revealed that select DNA repair enzyme activities in tumors impact response of therapeutic agents, suggesting a requirement for enzyme functional analyses to bolster precision medicine. Personalized or ‘Precision Prevention’ would provide an opportunity to identify those at high risk for a given disease so as to advise for targeted screening and primary prevention interventions to alter disease susceptibility [5]. DNA repair pathways maintain the integrity of the genome, help prevent the onset of cancer, disease and aging phenotypes [6] and play a significant role in the cellular and organismal response to environmental exposures [7–13]. As such, this critical requirement for DNA repair proteins and pathways in response to DNA damage implicates DNA repair proteins as prime targets for improving response to currently available anti-cancer regimens [14]. For example, inhibitors to several DNA repair proteins have been developed and are either undergoing clinical testing or are being considered for such [15–21]. Enzymes of the base excision repair (BER) pathway, including DNA glycosylases, endonucleases and end-trimming enzymes are over-expressed or mutated in many cancers, suggesting that defects in BER enzymes may play a role in cancer or disease following select environmental exposures. Conversely, since many BER enzymes are over-expressed in cancer cells and tumors, some may be essential for cancer growth or promote treatment resistance. For example, overexpression of the DNA glycosylase NEIL3 promotes both double-strand break repair and resistance to reactive oxygen species, suggesting a role for increased BER in drug resistance [22]. Identifying cells/tissues with functional alterations in this essential DNA repair pathway (BER) would be a first step in preventing disease as such ‘carriers’ would have an increased risk of exposure-associated pathology. Further, identifying essential cancer-dependent BER protein functions offers an opportunity for selective treatment options, such as seen with PARP1 inhibitors [23, 24]. Many of these potential cancer- or disease-specific DNA repair defects [25] or dependencies [26, 27] can be detected using current “omics” technologies. However, there are many enzyme defects, mutations that lead to changes in post-translational modifications or modulation of basal enzyme/activity levels that can only be detected from an analysis of protein-specific DNA repair function. Importantly, target validation, analysis and inhibitor development require functional assays. Thus, a technique directly measuring enzyme activity could offer a significant benefit for both enzyme inhibitor development and as a companion diagnostic.

Towards the design of a robust platform to assess the function of individual enzymes within DNA repair pathways as well as to define cellular/tumor DNA repair capacity, we have developed a sensitive and quantitative real-time assay that measures the activities of DNA BER enzymes. BER is the predominant mechanism for coping with spontaneous hydrolytic and oxidative DNA damage, and deficiencies in BER are associated with developmental problems, immune-dysfunction, cancer predisposition and neurological disease [28–32]. We recently reported the development of a molecular beacon assay to evaluate the activities of several BER enzymes that consists of a single strand of DNA with a single base lesion as the enzyme substrate [15, 33, 34]. Here, we describe the development of an enhanced fluorescent, quantitative DNA Repair Molecular Beacon (DRMB) plate-based assay that rapidly measures DNA repair enzyme activity. Further, the DRMB assay can be a companion diagnostic for the analysis of enzyme activity in cellular lysates and patient-derived samples to validate DNA repair inhibitor efficacy. We show that the DRMB assay is sensitive enough to detect alterations in the activities of DNA repair enzymes, including clinically relevant mutations in the major human apurinic/apyrimidinic (AP) endodeoxyribonuclease, APE1. To further extend the utility of this assay, a microbead-conjugated DRMB assay was developed to allow flow cytometric analysis. Our studies demonstrate the utility of this platform to quantitatively evaluate select DNA repair protein activities that would be useful to help guide precision cancer therapy options. In future efforts, the DRMB assay would be applicable for the discovery of DNA repair enzyme inhibitors and for the quantification of enzyme rates.

## RESULTS

### Optimization and validation of a plate-based DRMB assay

**Figure 1:**
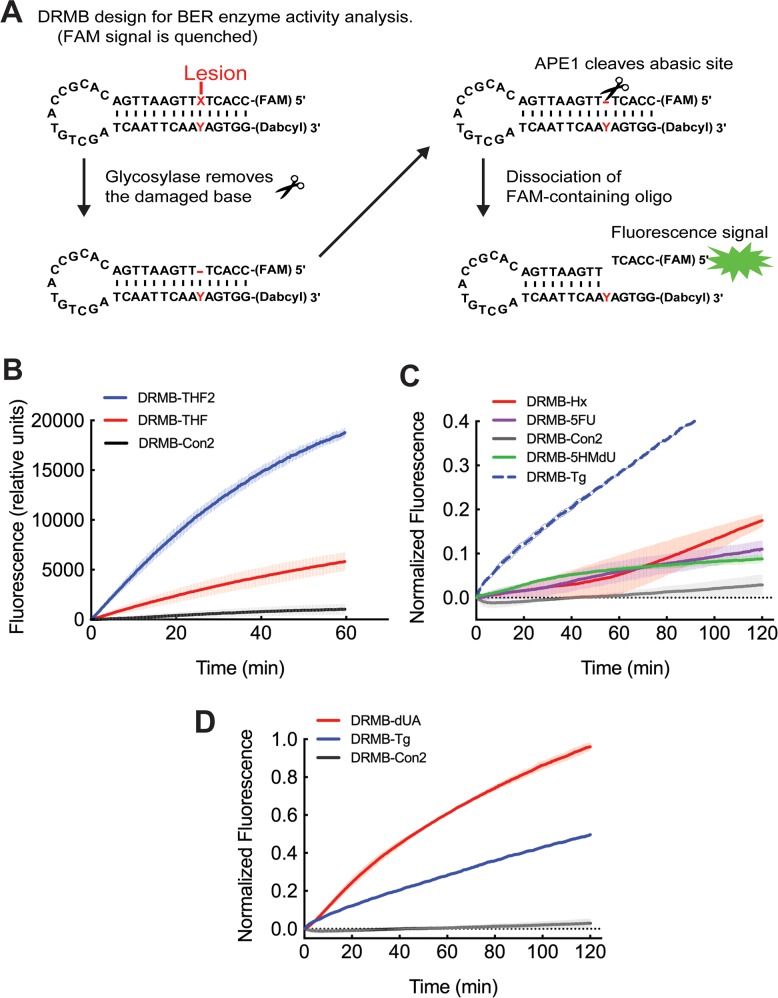
The new DNA repair molecular beacon (DRMB) design has increased sensitivity and enables measurement of several endogenous DNA repair protein activities with lesion specific molecular beacons **(A)** Diagram outlining the DNA Repair molecular beacon (DRMB) assay designed to evaluate BER enzyme activities. **(B)** Comparative analysis of the newly designed assay (DRMB-THF2) as compared to the beacon containing a guanidine adjacent to the 6-FAM fluorophore (DRMB-THF), which quenched the fluorescence signal. **(C)** LN429 (glioma) cell lysates probed for base lesion repair activity using DRMB assays containing the lesions Hypoxanthine (Hx, also known as inosine), 5-fluoro-uracil (5-FU) or 5-hydroxymethyl-2′-deoxyuridine (5-HMdU), as compared to a control beacon (DRMB-Con2) containing no DNA lesion. The plot for the DRMB assay containing the thymine glycol (Tg) lesion is also shown as a comparison to the plot in panel **(D)**. (D) LN429 (glioma) cell lysates probed for lesion repair activity using DRMB assays containing deoxyuridine (dU/A) or thymine glycol (Tg), as compared to a control beacon (DRMB-Con2) containing no DNA lesion. Plots (B, C, D) show the mean normalized fluorescence values of two independent experiments, each measuring activity in 3 wells, with error bars representing the range. Plot shown in (B) is in relative fluorescence units so as to be able to compare the two beacon designs. Plots in (C) and (D) show normalized fluorescence values as described in the Materials and Methods. Statistical parameters are also shown in Supplementary Tables 1 and 2.

The analysis of cell and tissue lysates with small changes in protein expression or modulation of enzyme activity due to somatic mutations or the result of inappropriate post-translational modification requires a sensitive and reproducible assay that can provide quantification of overall DNA repair capacity as well as details on enzyme rates. To address this need, we have developed an enhanced DRMB assay, as shown in Figure 1A, for the quantification of BER enzymatic activity. The overall time frame and assay workflow for the DRMB assay is shown in Supplementary Figure 1. The DRMBs have been engineered to form a stem-loop structure with a 13-nucleotide loop and a 15-base pair stem, conjugated with a 5′ 6-FAM fluorophore and a 3′ Dabcyl quencher moiety. In the original version of our molecular beacons [34], 6-FAM was conjugated to a 5′ guanosine. However, the conjugation of a fluorescent dye to a guanosine nucleotide can effectively quench the fluorescence signal via electron sharing/donor properties of the guanosine nucleotide [35]. Thus, we replaced the 5′ guanosine with a cytosine (Figure 1A) to increase the fluorescent signal and improve the signal-to-noise ratio greater than 3-fold. Beacons with the same structure, but no lesion, which should therefore not be processed by a DNA repair enzyme (DRMB-Con and DRMB-Con2, Table 1), served as negative controls. The complete details of all the DRMB substrates are defined in Table 1.

**Table 1:** Sequence and base modifications of the DRMB oligonucleotides

DRMB	5′ Modifier	Sequence 5′ -> 3′	Modified bases (X)	3′ Modifier	Target	Figure
Con	6-FAM	GCA CTATTG AAT TGA CAC GCC ATG TCG ATC AAT TCA ATA GTG C	-	Dabcyl	No damage control	1
Con2	6-FAM	CCA CTA TTG AAT TGA CAC GCC ATG TCG ATC AAT TCA ATA GTG G	-	Dabcyl	No damage control	1,2,3,5
THF	6-FAM	GCA CT**X**TTG AAT TGA CAC GCC ATG TCG ATC AAT TCA A**T**A GTG C	THF, terahydrofuran (Abasic site mimic); opposite a ‘T’	Dabcyl	APE1	1B
THF2	6-FAM	CCA CT**X** TTG AAT TGA CAC GCC ATG TCG ATC AAT TCA A**T**A GTG G	THF, terahydrofuran (Abasic site mimic); opposite a ‘T’	Dabcyl	APE1	1B, 2A, 3B, 3E, 4B, 5A, 6B
THF2/G	6-FAM	CCA CT**X** TTG AAT TGA CAC GCC ATG TCG ATC AAT TCA A**G**A GTG G	THF, terahydrofuran (Abasic site mimic); opposite a ‘G’	Dabcyl	APE1	2A
THF2/A	6-FAM	CCA CT**X** TTG AAT TGA CAC GCC ATG TCG ATC AAT TCA A**A**A GTG G	THF, terahydrofuran (Abasic site mimic); opposite a ‘A’	Dabcyl	APE1	2A
THF2/C	6-FAM	CCA CT**X** TTG AAT TGA CAC GCC ATG TCG ATC AAT TCA A**C**A GTG G	THF, terahydrofuran (Abasic site mimic); opposite a C’	Dabcyl	APE1	2A
8oxoG/C	6-FAM	CCA CT**X** TTG AAT TGA CAC GCC ATG TCG ATC AAT TCA A**C**A GTG G	8-oxoguanine (8-oxo-7,8-dihydro-2′-deoxyguanosine); opposite C	Dabcyl	OGG1	2B
8oxoG/A	6-FAM	CCA CT**X** TTG AAT TGA CAC GCC ATG TCG ATC AAT TCA A**A**A GTG G	8-oxoguanine (8-oxo-7,8-dihydro-2′-deoxyguanosine); opposite A	Dabcyl	OGG1	2B
C/8oxoG	6-FAM	CCA CT**C** TTG AAT TGA CAC GCC ATG TCG ATC AAT TCA A**X**A GTG G	C opposite 8-oxoguanine (8-oxo-7,8-dihydro-2′-deoxyguanosine)	Dabcyl	OGG1, MYH	2B
A/8oxoG	6-FAM	CCA CT**A** TTG AAT TGA CAC GCC ATG TCG ATC AAT TCA A**X**A GTG G	A opposite 8-oxoguanine (8-oxo-7,8-dihydro-2′-deoxyguanosine)	Dabcyl	OGG1, MYH	2B
Tg/A	6-FAM	CCA CT**X** TTG AAT TGA CAC GCC ATG TCG ATC AAT TCA A**A**A GTG G	Thymine glycol (5,6-dihydroxy-5,6-dihydrothymine); opposite A	Dabcyl	NTH1, NEIL1	1D, 3F
5FU	6-FAM	CCA CT**X** TTG AAT TGA CAC GCC ATG TCG ATC AAT TCA A**A**A GTG G	5-fluoro-uracil (5FU); opposite A	Dabcyl	UNG, SMUG1, TDG	1C
5HMDU	6-FAM	CCA CT**X** TTG AAT TGA CAC GCC ATG TCG ATC AAT TCA A**A**A GTG G	5-hydroxymethyl-2′-deoxyuridine; opposite A	Dabcyl	SMUG1	1C
Hx	6-FAM	CCA CT**X** TTG AAT TGA CAC GCC ATG TCG ATC AAT TCA A**T**A GTG G	Hypoxanthine (Inosine); opposite T	Dabcyl	MPG	1C, 5C
dU/A	6-FAM	CCA CT**X** TTG AAT TGA CAC GCC ATG TCG ATC AAT TCA A**A**A GTG G	deoxyU (deoxyUridine); opposite A	Dabcyl	UNG, SMUG1	1D, 3C, 5B
Biotin-THF	Iowa Black^®^ Dark	GGA CT**T**TTG AAT TGA CACGCC AT(-Biotin)G TCG ATC AAT TCA A**X**AGTCC	THF, terahydrofuran (Abasic site mimic); opposite a ‘T’	6-FAM	APE1	6B, 6D, 6F

Many different nucleases in cellular protein extracts may degrade the DNA beacon. We therefore aimed at reducing background due to non-specific release of the fluorescence signal or quencher and so the reaction buffer was supplemented with 0.5 mM EDTA to chelate free Mg^2+^, a metal co-factor required by many cellular nucleases. As shown in Supplementary Figure 2A, with the supplement of Mg^2+^, the negative control DRMB-Con2 was quickly degraded by the exonuclease activity of purified recombinant His-APE1 (or a contaminant), and 53% of the maximum fluorescent signal was released at the beginning of the assay and reached 95% at the end of the assay. In contrast, without the supplement of Mg^2+^, DRMB-Con2 was not hydrolyzed by the His-APE1 preparation (0.37% of the maximum fluorescent signal at the end of the assay). When DRMB-THF2, the improved substrate for APE1 endonuclease activity, was used in the DRMB assay, the supplementation of Mg^2+^ resulted in the release of 87% of the maximum fluorescent signal at the start of assay. Such a high background due to non-specific nuclease activity would render the DRMB assay system impractical (Supplementary Figure 2B). This observation is consistant with an earlier report suggesting that APE1, in the presence of Mg^2+^, can hydrolyze 3′ or 5′-linked FAM or dabcyl moieties [36]. Thus, we used the reaction buffer containing 0.5 mM EDTA throughout the remainder of the analyses.

Inter- and intra-experimental variation that stem from dissimilarities in beacon input or beacon labelling efficiency or stability is also reduced by the normalization of the fluorescence signal to the maximum fluorescence signal of each well after full denaturation = Fl(T_max_). Details on the normalization protocol are described in the Materials and Methods, and previously [34]. As shown in all the activity assays, none of the control beacons showed any measurable DNA cleavage activity, thereby demonstrating low background activity and strong signal specificity with the defined reaction conditions and buffer preparation.

In order to test the impact of the modification to the assay, we began with a DRMB engineered with a tetrahydrofuran (THF) lesion that mimics an AP site [37] and is a robust substrate for the BER endodeoxyribonuclease APE1 (Figure 1B) (DRMB-THF). The re-designed DRMB (DRMB-THF2, 5′ guanosine replaced by a cytosine) is significantly improved as compared to the original, revealing a >350% increase in fluorescence signal (p<0.005 for k; p<0.0001 for Y_max_) (Figure 1B) [34]. This advanced assay greatly improves the sensitivity of the DRMB assay for the measurement of cellular changes in DNA repair capacity. Therefore, we next evaluated the assay in cell lysates using DRMB assays with a variety of lesions that would be a substrate for BER glycosylases (Table 1) [38].

### DRMB assay reveals differential lesion-specific BER activity

BER is initiated by one of eleven DNA glycosylases (Table 2). Each is expressed in the nucleus with several reported to localize in the cytosol and/or the mitochondria (Table 2). Glycosylases can be divided into either mono-functional (e.g., MPG) or bi-functional (e.g., OGG1) designations, with the latter involving both base lesion removal activity in combination with either an associated β-elimination or β,δ-elimination function to cleave the DNA backbone (see Table 2 and references within). Whereas some DNA glycosylases have unique substrate specificities, there is considerable overlap in lesion recognition and removal for several of the enzymes (Table 3). It has been suggested that some of the glycosylases function as backup enzymes to ensure robust BER capacity and prevent disease onset, such as the demonstration that SMUG1 acts to complement UNG to protect from cancer formation in MSH2 deficient model systems [39]. As such, an assay such as this that measures lesion repair may have broad applicability when enzyme complementation is of concern.

**Table 2:** Human DNA glycosylases

**Human Mono-functional DNA Glycosylases**					
**Gene Symbol**	**Gene Name**	**Gene ID**	**Uniprot Accession Number**	**Organelle expressed**	**Citation**
MBD4	Methyl-CpG binding domain protein 4	8930	O95243	Nucleus	[90, 91]
MPG	N-methyl DNA glycosylase	4350	P29372	Cytoplasm	[91]
				Nucleoplasm	[92]
MUTYH (MYH)	mutY homolog (E. coli)	4595	Q9UIF7	Nucleoplasm	[92]
				Nucleus	[93]
				Mitochondrion	[94]
UNG	Uracil DNA glycosylase	7374	P13051	Nucleolus	[95]
				Mitochondria	[95]
SMUG1	Single-strand-selective mono-functional uracil-DNA glycosylase 1	23583	Q53HV7	Nucleolus	[95]
TDG	Thymine DNA glycosylase	6996	Q13569	Nucleoplasm	[92]
**Human Bi-functional DNA Glycosylases (with associated β-elimination)**					
**Gene Symbol**	**Gene Name**	**Gene ID**	**Uniprot Accession Number**	**Organelle expressed**	**Citation**
OGG1	8-oxoguanine DNA glycosylase	4968	O15527	Nucleoplasm	[92]
				Mitochondrion	[94]
NEIL3	nei endonuclease VIII-like 3	55247	Q8TAT5	(Suspected nuclear)	
NTHL1 (NTH1)	nth endonuclease III-like 1	4913	P78549	Nucleoplasm	[92]
				Mitochondrion	[94]
				Nucleus	[96]
**Human Bi-functional DNA Glycosylases (with associated β,δ-elimination)**					
**Gene Symbol**	**Gene Name**	**Gene ID**	**Uniprot Accession Number**	**Organelle expressed**	**Citation**
NEIL1	nei endonuclease VIII-like 1	79661	Q96FI4	Nucleus	[97]
				Cytoplasm	[97]
NEIL2	nei endonuclease VIII-like 2	252969	Q969S2	Nucleus	[98]
				Cytoplasm	[98]

**Table 3:** Mammalian DNA glycosylases substrates

Gene Symbol	Reported Substrate	Citation
MBD4	U or T in U/TpG; 5-meCpG	[38]
	5-formyluracil; 5-(hydroxymethyl)-U	[99]
	Tg:G	[100]
MPG (AAG)	3-meA; 7-meA; 3-meG; 7-meG; hypoxanthine; ethenoA; ethenoG	[38, 101–103]
	1,N2-εG:C; U:G; ethanoadenine; 1-methylguanine	[104]
	etheno-A(ss); hypoxanthine(ss); ssU	[104]
	8-oxoG:C (Mouse)	[105]
	cyanuric acid:CT>GA	[99]
MUTYH (MYH)	A:G; A:8-oxoG; C:A; 2-OH-A	[38]
	8-oxoA:G	[106]
NEIL1	TgG; 5-OH-C; 5-OH-U:AT>G	[38]
	guanidinohydantoin; iminoallantoin; spiroiminodihydantoin	[107]
	5,6-dihydro-T; 5,6-dihydro-U:G/C/A>T; fapyG:C; 8-oxo-G:C/G>T>A; fapyA:T	[99]
	8-oxo-A:C	[108]
NEIL2	5-OH-U:G>T>A; 5-OH-C	[38]
	5,6-dihydro-U:G/A; 8-oxo-G:C/A; 5,6-dihydrothymine	[99]
	Guanidinohydantoin; iminoallantoin	[107]
NEIL3	spiroiminodihydantoin (Sp):C; guanidinohydantoin (Gh):C	[109]
	Tg; FapyA; FapyG; 5-OH-U; 5-OH-C	[109]
NTHL1 (NTH1)	T or C-glycol; FapyA	[38]
	5,6-dihydro-U:G/A; 5-formyl-U; 5,6-dihydroxy-C; 5,6-dihydro-T	[99]
	urea; 5-hydroxy-5,6,-dihydro-T; 5-OH-U:G; 5-OH-C:G>A	[99]
	8-oxoG:G	[110]
OGG1	8-oxoG:C/T/G; me-FapyG:C; FapyG:C	[38]
	8-oxoA:C	[111]
	urea	[99]
SMUG1	ssU; U:G; U:A	[38]
	5-fluorouracil:G; 5-chlorouracil:G; 5-carboxyuracil:G	[112]
	5-formyl-U; 5-hydroxyuracil	[99]
	5-(hydroxymethyl)-U	[99, 113]
TDG	U:G; T:G; ethenoC:G	[38]
	5-fluorouracil; 5-hydroxymethyluracil; εC:A; hypoxanthine:G; 5-bromouracil	[114]
	5-formyl-U	[99]
	Tg:G	[100]
	7,8-dihydro-8-oxoadenine (8oxoA)/T	[111, 115]
UNG	ssU; U:G; U:A; 5-fluorouracil	[38]
	5,6-dihydroxy-U:G	[99]
	5-OH-U:G	[99, 116]
	Isodialuric acid; Alloxan	[116]

To test the DRMB assay for the ability to measure base damage removal, we designed assays with base lesions that would be representative of many of the major BER glycosylase activities (Tables 1, 3). Our initial vetting of the DRMB assays utilized nuclear lysates from the glioma cell line LN428, since we find that these cells express all 11 DNA glycosylases albeit at varying levels (Supplementary Figure 3). As might be predicted from the variation in glycosylase expression levels, we find drastic variation in, as well as unique apparent kinetic profiles for, the cellular capacity to remove specific base lesions, including hypoxanthine (Hx, also known as inosine), 5-fluoro-uracil (5-FU), 5-hydroxymethyl-2′-deoxyuridine (5-HMdU), deoxyuridine (dU) and thymine glycol (Tg) (Figure 1C, 1D). Based on these findings, our data show that the DRMB assay is applicable for a variety of relevant base lesions (see Supplementary Tables 1 and 2 for statistical values).

Hx is a substrate primarily for the mono-functional methylpurine DNA glycosylase, MPG [38], with a low level of activity by TDG (Table 3). We find that in cell lysates from LN428 cells, there is a weak but measurable capacity to remove the Hx lesion (Figure 1C), with little or no fluorescent signal from the control DRMB (DRMB-Con2). This is consistent with the relatively low expression of MPG, as we have shown (Supplementary Figure 3 and [15]). Given the elevated levels of TDG mRNA in LN428 cells, these functional analyses tend to support MPG as the primary enzyme involved in Hx repair. Moreover, excision/incision of the beacon containing the 5-FU lesion is quite similar to that of the beacon containing the 5-HMdU lesion (Figure 1C). The 5-FU lesion is known to be a substrate for MBD4, NTH1, SMUG1 and TDG (Table 3), whereas the 5-HMdU base lesion is reported as a substrate only for MBD4, SMUG1 and TDG (Table 3). Given the high levels of expression of all of these enzymes in LN428 cells (Supplementary Figure 3), it is surprising that the excision rates are low and points to the benefit of an activity assay when evaluating overall lesion repair capacity as opposed to an analysis of mRNA levels. Conversely, the excision of dU and Tg was robust (Figure 1D), and the mRNA levels in LN428 cells for the major enzymes involved in the excision of these lesions [UNG (dU/A) as well as NTH1 and TDG (Tg)] are also high (Supplementary Figure 3). We show here that utilization of the DRMB assay allows one to measure overall base lesion removal capacity, regardless of the BER enzymes responsible. Defining overall base lesion removal (excision/repair) capacity may have a greater correlation to cellular capacity for response to select DNA damaging agents or the onset of disease than evaluating mRNA or protein expression levels of individual repair enzymes.

### Lesion context determines BER activity as defined by the DRMB assay

**Figure 2:**
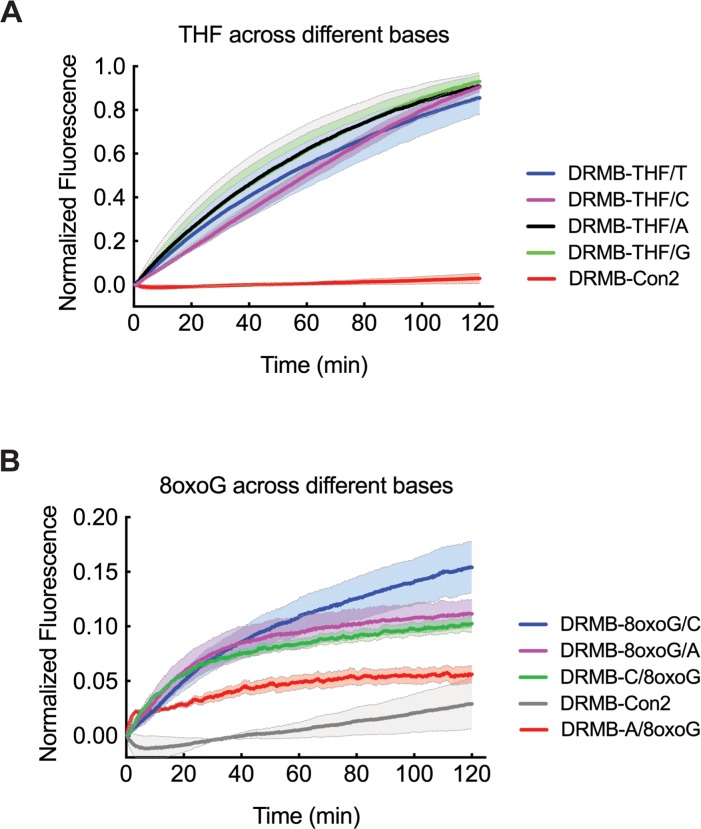
Increased sensitivity of the DRMB assay reveals subtle differences in incision or excision rates dependent on the base opposite the lesion LN429 (glioma) cell lysates probed for lesion repair activity using **(A)** DRMB-THF2 assays designed with each substrate containing a different base (T, C, G, A) opposite the lesion or **(B)** DRMB-8oxoG assays designed with the 8oxoG on either strand and with either a C-base or an A-base opposite the 8oxoG lesion. In each, activity is as compared to a control beacon (DRMB-Con2) containing no DNA lesion. Plot data show normalized fluorescence values and are the mean of two independent experiments; error bars report the range of the mean values of the independent experiments. Statistical values are listed in Supplementary Tables 1 and 2.

Previous enzymatic characterization of APE1-mediated cleavage of DNA oligonucleotides with a THF lesion suggested that the base opposite the lesion (THF) had no discernable impact on activity, as measured using the classical gel-based assay [40]. Conversely, the flanking bases had a significant impact on APE1 activity, particularly if it was a mismatch [40, 41], likely due to increased substrate flexibility that is favorable to the active site [42]. We reasoned that with our enhanced analytical assay, we might observe subtle variations in activity or rates depending on the base (T, C, A, G) or base-type (purine or pyrimidine) opposite the THF lesion. We therefore compared the hydrolysis of the THF lesion opposite each of the 4 bases (T, C, A, G) using lysates from the glioma cell line LN428 that has robust APE1 activity. As shown (Figure 2A), the DRMB assay was able to depict some variation in APE1 activity that depends on the base opposite the THF lesion, with a trend towards a lower rate of incision for substrates with a pyrimidine opposite the AP site relative to a purine. The overall activity at THF/C was indeed 15% lower from that of THF/A or THF/G (Supplementary Table 2) and the incision rates differed significantly at THF/C beacons (p<0.001, Supplementary Table 1).

Next, we evaluated the repair (base lesion removal) of 8-oxo-7,8-dihydro-2′-deoxyguanosine (8-oxoguanine, 8-oxoG), a mutagenic base byproduct that occurs as a result of exposure to reactive oxygen species (Figure 2B). For this analysis, to assess sequence context specific repair, we developed 4 unique DRMB assays, allowing an analysis of removal of 8-oxoG when across from a C base or an A base and each designed on either strand (see Table 1). The 8-oxoG lesion is the most extensively studied oxidative lesion of guanine and is primarily repaired by the DNA glycosylase enzyme OGG1, particularly when opposite a C (Table 3). However, there are reports of 8-oxoG lesion removal by MPG (in mouse) and by NEIL1, NEIL2 and NTH1 in humans (Table 3). Interestingly, using our DRMB assay, we show that repair kinetic parameters (k, Y_max_) of the 8-oxoG lesion differ significantly depending on the opposite base and this also varies depending on the 8-oxoG containing DNA strand (Figure 2B, Supplementary Tables 1 and 2). Overall, we find that the removal of 8-oxoG is most robust when opposite the C base.

If the 8-oxoG lesion is not repaired prior to replication, there is an increase in mutations, mostly G to T transversions, that arise from DNA synthesis past the lesion and insertion of dAMP opposite 8-oxoG by the replicative polymerases. To avoid deleterious G to T transversion mutations, the 8-oxoG/A mis-pair can be selectively corrected by another round of BER, this time initiated by MUTYH [43]. Using a DRMB substrate that should be selective for MUTYH (A/8oxoG), we observe a low level of excision capacity (Figure 2B), in-line with the expression levels of MUTYH in these cells (Supplementary Figure 3). We note that for the mono-functional DNA glycosylases (i.e. MPG, UNG, SMUG1), which only excise the substrate base, but leave the DNA backbone intact, AP site processing, and thus fluorescent signal generation, likely takes place due to efficient endogenous APE1 incision (Figure 1B), despite the presence of EDTA in the reaction buffer, consistent with earlier cellular studies [44].

### Molecular beacon assay for BER is dependent on APE1 activity

**Figure 3:**
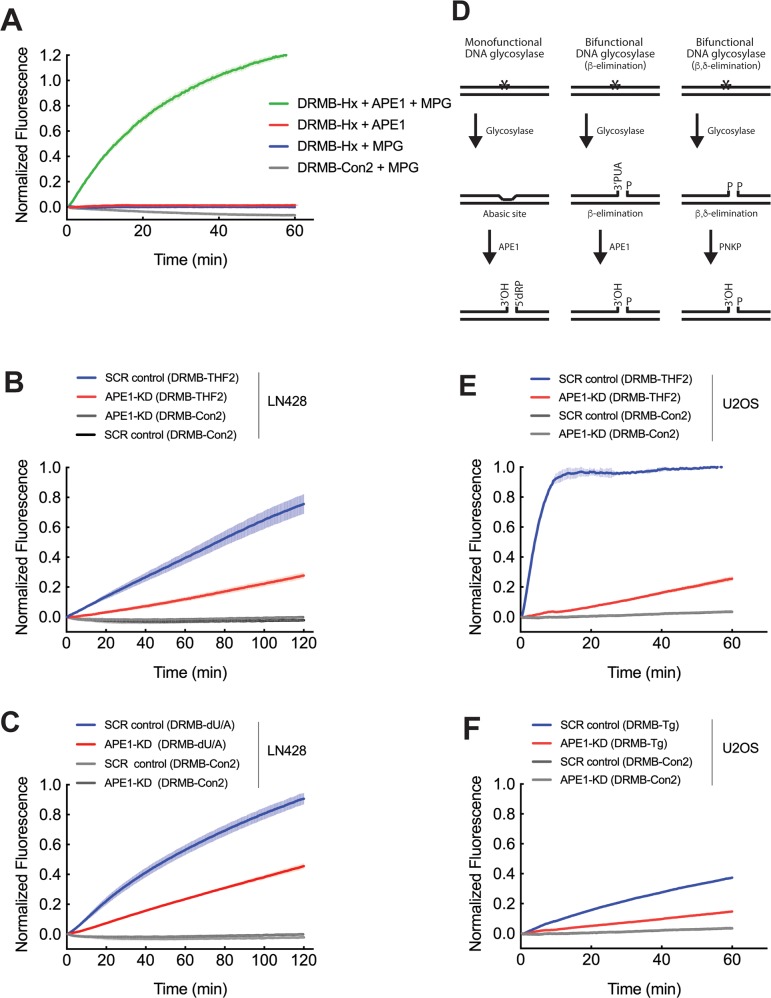
Both mono-functional and bi-functional DNA glycosylases depend on APE1 for DNA backbone cleavage in cell lysates **(A)** Purified methylpurine DNA glycosylase (MPG) and APE1 probed for lesion repair activity using DRMB-Hx and DRMB-Con2 assays. **(B)** Analysis of APE1 activity: LN429/SCR (LN429 cells expressing a scrambled control shRNA) and LN429/APE1-KD cell lysates probed for lesion repair activity using DRMB-THF2 assays, as compared to a control beacon (DRMB-Con2) containing no DNA lesion. **(C)** Analysis of uracil glycosylase activity: LN429/SCR (LN429 cells expressing a scrambled control shRNA) and LN429/APE1-KD cell lysates probed for lesion repair activity using DRMB-dU/A assays, as compared to a control beacon (DRMB-Con2) containing no DNA lesion. **(D)** Simplified schematic detailing the lesion removal and strand cleavage activities of mono-functional and bi-functional DNA glycosylases and APE1 in BER. Mono-functional DNA glycosylase activity (Left) leaves an abasic site as a substrate for APE1, resulting in DNA strand cleavage. Bifunctional DNA glycosylases excise the base and then catalyze either (Center) β-elimination, resulting in a 3’phospho, unsaturated aldehyde (PUA) and a 5’phosphate (P) in the gap that is then further processed by APE1. (Right) β,δ-elimination resulting in a 3’phosphate (P) and a 5’phosphate (P) in the gap that can then be further processed by polynucleotide kinase 3′-phosphatase (PNKP). **(E)** Analysis of APE1 activity: U2OS/SCR (U2OS cells expressing a scrambled control shRNA) and U2OS/APE1-KD cell lysates probed for lesion repair activity using DRMB-THF2 assays, as compared to a control beacon (DRMB-Con2) containing no DNA lesion. **(F)** Analysis of glycosylase activity for removal of thymine glycol: U2OS/SCR (U2OS cells expressing a scrambled control shRNA) and U2OS/APE1-KD cell lysates probed for lesion repair activity using DRMB-Tg assays, as compared to a control beacon (DRMB-Con2) containing no DNA lesion. Plot data show normalized fluorescence values and are the mean of three independent experiments with error bars representing SEM. Statistical values are listed in Supplementary Tables 1 and 2.

BER is used by cells to repair small, non-helix-distorting base lesions, and APE1 is the major endonuclease to hydrolyze the DNA backbone at the AP site following removal of a damaged base by a DNA glycosylase [38, 45]. The readout from the DRMB assay should mimic this mechanism, in which a glycosylase is required to remove the lesion in question. However, no signal would be detected until cleavage of the DNA strand at the resulting AP site by APE1, which would drive dissociation of the fluorophore from the quencher and the generation of the fluorescent signal (Figure 1A). A DRMB assay with a substrate containing a lesion preferentially repaired by methylpurine DNA glycosylase (MPG), together with purified, recombinant human APE1 (Supplementary Figure 4A), allows us to assess this sequential activity. As shown in Supplementary Figure 4B, purified APE1 could effectively cleave its cognate THF containing substrate (DRMB-THF2). There was no fluorescent signal when a non-lesion control beacon was evaluated (DRMB-Con2), which indicated that there was no random cleavage by APE1 on either DRMB (Supplementary Figure 4B). In support of the specificity of the DRMB assay, when an Hx-beacon (DRMB-Hx), which is not a substrate for APE1, was incubated with purified APE1, there was no increase in fluorescent signal (Figure 3A, red line). When MPG was added to the reaction mixture, DRMB-Hx, which is a known substrate of MPG [34, 38], was processed by the combination of enzymes, resulting in a strong fluorescence signal (Figure 3A, green line). This result is consistent with APE1 cleavage of the AP site product following MPG-mediated base lesion removal (Figure 3A). No fluorescence signal was detected in the reaction with MPG alone (Figure 3A, blue line), indicating that cleavage of the AP site by APE1 is necessary to release the fluorophore from the quencher.

Whereas the fluorescent readout of the DRMB-Hx assay is dependent on both base cleavage by the mono-functional DNA glycolyase (MPG) and DNA strand hydrolysis mediated by APE1 when using purified proteins (Figure 3A), our ultimate goal is to use the DRMB assay for the analysis of cell and tissue lysates. We therefore next asked if the DRMB assay, when used with human cell lysates, is also dependent on APE1, since there are many nucleases in the cell, including the related enzyme APE2 that may function in PCNA-dependent BER [46]. For this analysis, we established two viable stable APE1 knockdown (APE1-KD) cell lines using lentiviral shRNA targeting APE1 in LN428 glioblastoma and in U2OS osteosarcoma cells. As shown, the mRNA of *APE1* in LN428/APE1-KD cells was reduced to about 5-10% of that of the scramble control cells (SCR) and was reduced to as low as 2% in U2OS/APE1-KD cells (Supplementary Figures 4C and 4D). The level of APE1 protein was reduced as a result of the depletion of *APE1* mRNA, which was verified by immunoblotting analysis in nuclear lysate preparations (Supplementary Figures 4E and 4F).

Nuclear lysates from the viable LN428/APE1-KD cell lines were prepared and used to assess the impact of APE1 depletion on the hydrolysis of the THF containing beacon (DRMB-THF2). Supportive of the depletion of APE1 and selectivity of the DRMB-THF2 assay for APE1 function, cleavage activity of DRMB-THF2 in the LN428/APE1-KD cell lysate was significantly decreased to ~32% (AUC) of the SCR control lysate (p<0.005, Supplementary Tables 1 and 2) (Figure 3B). To further confirm that APE1 is the major protein to cleave the AP site generated from the removal of a damaged base by a mono-functional DNA glycosylase, we evaluated the change in uracil DNA glycosylase activity, primarily carried out by UNG, the primary DNA glycosylase with specificity for uracil in double-stranded DNA [33, 38] (Table 3). Uracil DNA glycosylase activity was evaluated using a dU/A containing beacon (DRMB-dU/A) in control cells and after depletion of APE1 (Figure 3C). As shown, the rate of uracil removal activity detected was significantly reduced in the cell lysate from the LN428/APE1-KD cells (p<0.0001, Supplementary Tables 1 and 2), consistent with the reduced DRMB-THF2 activity in these APE1-KD cells. This is also supportive of a major role for APE1 in DNA strand cleavage following base lesion removal by a mono-functional DNA glycosylase (Figure 3D) [38].

### DRMB-THF2 assay reveals APE1 dependence of bi-functional glycosylases affecting net BER efficiency

Whereas mono-functional DNA glycosylases can only remove the damaged base, requiring APE1 to hydrolyze the DNA backbone [38], bi-functional DNA glycosylases, such as OGG1, NTH1 and NEIL3, can remove the lesion and subsequently cleave the DNA backbone through a β- or β,δ-elimination step [38], as depicted in Figure 3D. Bi-functional DNA glycosylases excise the base and then catalyze either β-elimination, which results in a 3’phospho, unsaturated aldehyde (PUA) and a 5’phosphate (P) in the gap that is further processed by APE1, or β,δ-elimination, which generates a 3’phosphate (P) and a 5’phosphate (P) in the gap that can be processed by polynucleotide kinase 3′-phosphatase (PNKP) (Figure 3D). As such, bi-functional glycosylases can create a single-strand break without the need for APE1-mediated endonuclease activity [38]. However, it has been suggested that the dominant mechanism for some bi-functional DNA glycosylases may be mono-functional base hydrolysis [47, 48]. We tested the incision activity of such glycosylases by measuring activities of NTH1 using a Tg-containing substrate, since we recently found that the majority of Tg lesion removal is dependent on NTH1 activity [49]. Towards this end, we measured Tg lesion processing using lysates from the U2OS/APE1-KD cell line with a strongly decreased APE1 activity (Figure 3E). Here, we find a significant reduction (>3-fold) of the incision rate (p<0.0001, Supplementary Tables 1 and 2) in the cell lysate from U2OS/APE1-KD cells as compared to U2OS/SCR cells (Figure 3F, Supplementary Table 2). It was surprising that the activity of NTH1 (as assessed using a Tg-containing substrate), a bi-functional DNA glycosylase, was negatively affected by the depletion of APE1. Thus, enabled by the DRMB assay, our results indicate that the DNA repair functions of both mono-functional and bi-functional DNA glycosylases are affected by APE1 presence and/or activity and support the previous suggestion that most DNA glycosylases operate as a mono-functional enzyme [47, 48].

### Activity alterations in somatic and germline APE1 mutants as measured with the DRMB-THF2 assay

**Figure 4:**
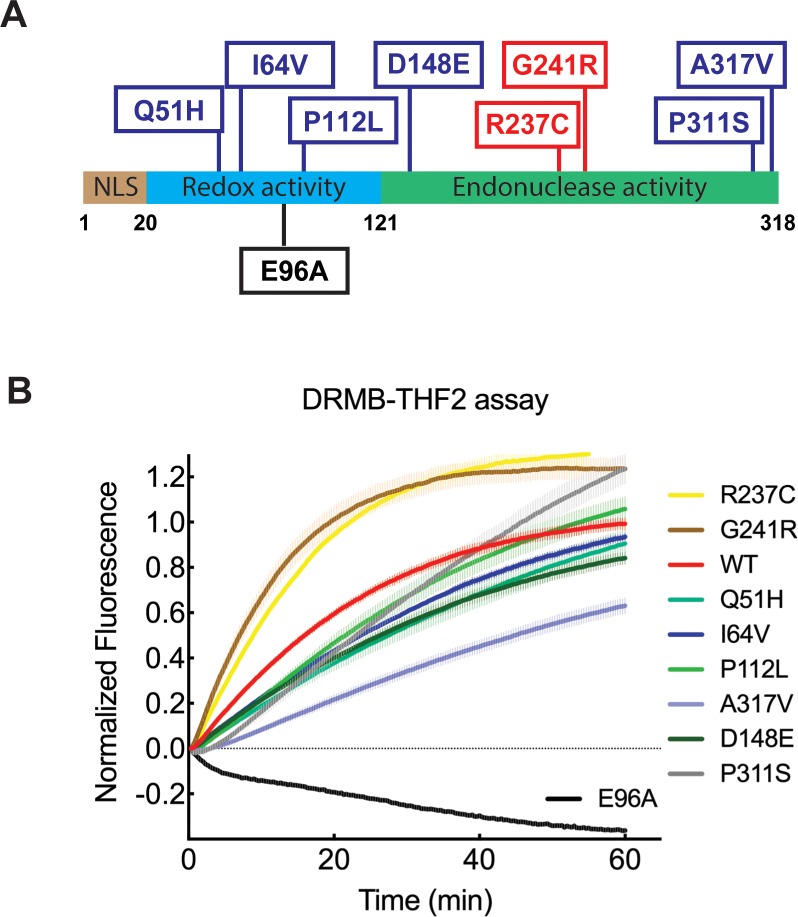
DRMB assay detected functional changes of APE1 with single amino acid substitutions **(A)** The position of the nine single amino acid substitutions of APE1 is indicated in the diagram. The red boxes indicate mutants with increased initial rate activity as determined by the DRMB-THF2 assay. The blue boxes indicate mutants with decreased initial rate activity as determined by the DRMB-THF2 assay. The black boxes indicate a mutant with a loss of activity as determined by the DRMB-THF2 assay. **(B)** APE1 mutants and WT protein (0.5 μg each) were analyzed for abasic site cleavage activity using the DRMB-THF2 assay. The activity of each protein was normalized to the maximum fluorescent signal within each well, Fl(T_max_), as described in the Materials and Methods and previously [34] and then normalized to each APE1 protein concentration detected by immunoblotting analysis using an APE1 antibody. Plots show the mean of three independent experiments, with error bars representing SEM. Statistical values are listed in Supplementary Tables 1 and 2.

The cytotoxic activity of some chemotherapeutic agents and, in part, of radiotherapy, is based on the generation of DNA damage, such as AP sites generated through BER at base lesions. APE1 is a vital protein in the BER pathway, recognizing and facilitating the repair of abasic lesions known to act as blocks to transcription or replication, or lead to mutations or telomere loss [50–53]. A robust and sensitive activity assay for APE1 is therefore crucial to predict tumor responses and identify patients that may respond to different treatments. We have shown that the DRMB-THF2 assay is sufficiently sensitive to differentiate the activity of APE1 in cell lysates when comparing changes in expression levels (Figure 3B, 3E). An important next step is to evaluate the changes in activity that might derive from inherited or somatic mutations in the *APE1* gene [54–60]. To further interrogate the sensitivity of the DRMB-THF2 assay, we measured the activities of purified wild type (WT) APE1 protein, a targeted active site mutant (E96A) and eight missense mutants (Figure 4A) in our earlier defined reaction conditions with EDTA. As shown in Figure 4B, compared to APE1(WT), the APE1 mutants G241R and R237C showed an increase in the initial rate of activity, whereas the APE1 mutants A317V, D148E, Q51H, I64V showed suppression of THF-cleavage activity (see Supplementary Tables 1 and 2 for statistical values). The APE1 E96A mutation essentially abolished the activity of APE1, which is consistent with an earlier report [61]. Thus, the DRMB assay allows the identification of activity-changing mutations.

### Molecular Beacons detected varying rates of repair capacity in leukemia cells and normal human peripheral blood mononuclear cells

**Figure 5:**
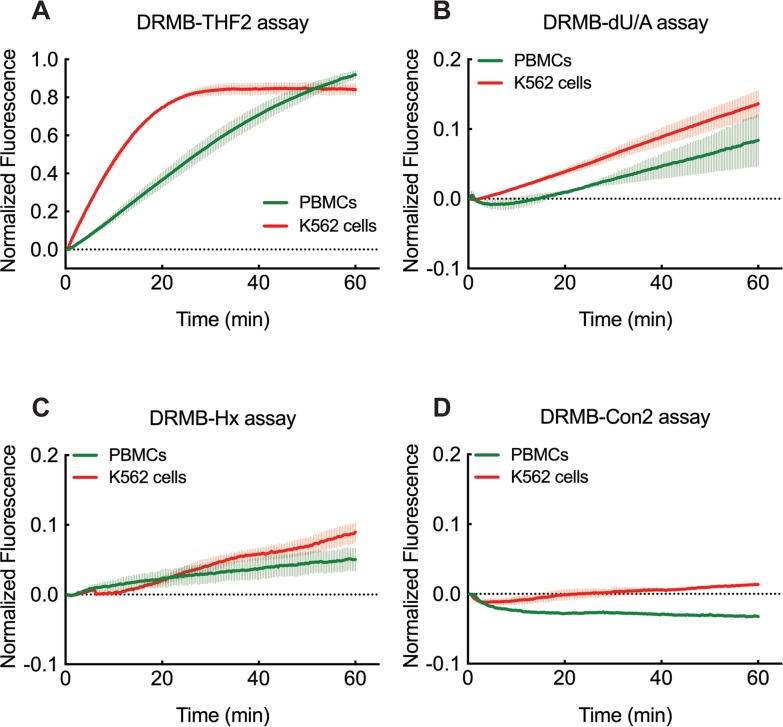
DRMB assay for the analysis of base lesion removal in K562 leukemia cells and normal human PBMCs DNA lesion repair activities measured by DRMB assays for **(A)** THF, **(B)** dU, or **(C)** Hx and compared to a control DRMB assay with no lesion **(D)** and using lysates (ultrasonication method) from PBMCs and K562 leukemia cells as indicated. Plots show the mean of two independent experiments, with error bars representing the range. Statistical values are listed in Supplementary Tables 1 and 2.

The mechanism of many chemotherapy drugs and radiotherapy is to induce cancer cell death by damaging the DNA. By extension, the toxicity to those treatments is also dependent, in part, on the DNA repair capacity of the cells. It has been suggested that cancer cells, especially cancer stem cells, have higher DNA repair capability compared to normal cells [62]. Also, DNA repair activities or their inhibition may vary in normal tissues of individuals [63, 64]. We therefore tested whether DNA repair activities of APE1, UNG and MPG are measurable in freshly isolated peripheral blood mononuclear cell (PBMCs) and in K562 leukemia cells using the DRMB-THF2, DRMB-dU/A and DRMB-Hx assays. The K562 human chronic myelogenous leukemia cell line shows higher activity of APE1 (Supplementary Table 2), and a slight increase in the removal of uracil and hypoxanthine, activities primarily conducted by UNG and MPG (Figure 5A–5C, Supplementary Table 2). Control DRMB assays, as we have seen in all other analyses, did not show an increase in signal (Figure 5D). Slight variations in repair function are visible in PBMCs, highlighting the power of the DRMB assay to reveal potential cell specific BER activity differences across cell types.

### Application of DRMBs as an end-point measurement tool using flow cytometry

**Figure 6:**
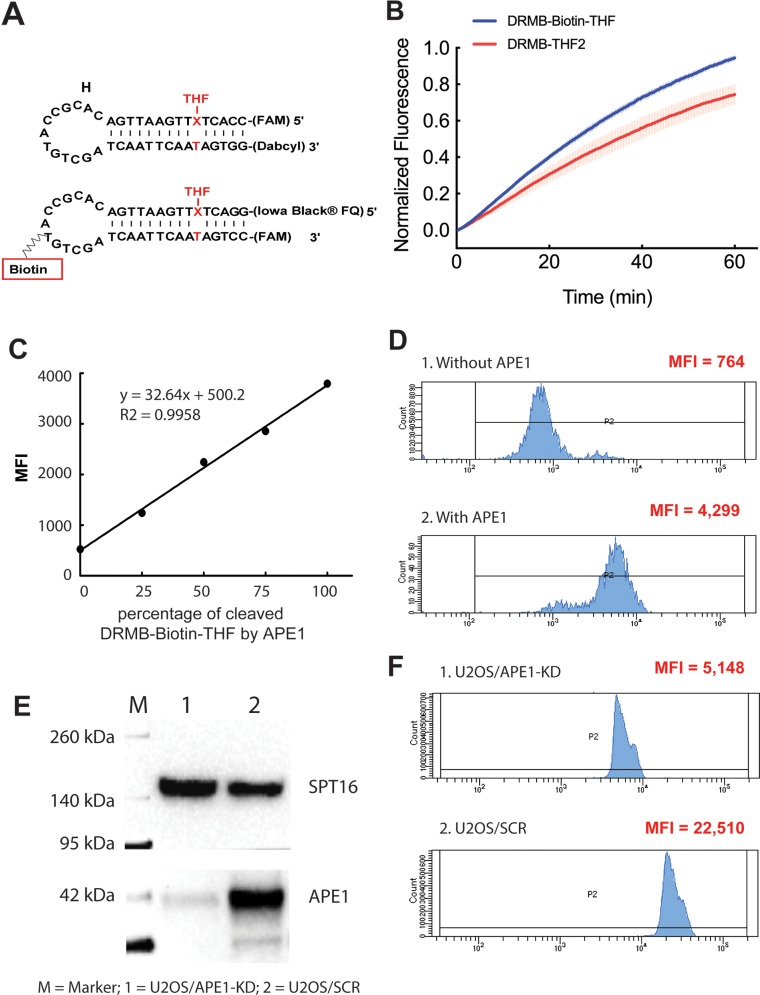
Evaluation of APE1 activity using a DRMB-Bead assay via flow cytometry analysis **(A)** Diagram of the DRMB-Biotin-THF with a T at the loop region of the DRMB-THF2 assay replaced with a biotin labeled T to allow conjugation to streptavidin beads. Further, the CC-FAM at the 5′ end of the DRMB-THF2 was replaced with GG-Iowa Black^®^ Dark quencher and the GG-Dabcyl at the 3′ end was replaced with a CC-FAM. Thus, after cleavage of beacon and release of the quencher, the fluorescent signal (FAM) will remain on the beads for signal measurement by flow cytometry. **(B)** Purified APE1 protein probed for abasic site cleavage activity using DRMB-THF2 and DRMB-Biotin-THF assays in solution.Plot data are the mean of three independent experiments, with error bars representing SEM. **(C)** Mixing fully cleaved and un-cleaved DRMB-Biotin-THF formed different ratios of fully cleaved and un-cleaved DRMB-Biotin-THF beacons. After those beacon mixtures were captured by streptavidin beads and analyzed by flow cytometry, the ratio of fully cleaved and un-cleaved DRMB-Biotin-THF were then differentiated by the Geo mean value. Data is represented as MFI (mean fluorescence intensity-geometric mean) of each mixture of beacons, R^2^ value = 0.9958. **(D)** Flow cytometry scan of the DRMB-Biotin-THF bound to beads and after incubation in buffer (1: without APE1) or with added APE1 (2: with APE1). Data is represented as MFI (mean fluorescence intensity-geometric mean). **(E)** Immunoblots showing the depletion of APE1 protein in U2OS/APE1-KD cell lysate compared to that of the U2OS/SCR control. **(F)** Flow cytometry scan of the DRMB-Biotin-THF bound to beads and after incubation in cell lysate (1: U2OS/APE1-KD or 2: U2OS/SCR). Data is represented as MFI (mean fluorescence intensity-geometric mean).

Since flow cytometry can analyze any fluorescent particle in the proper size range, we designed a bead-conjugated Molecular Beacon system (DRMB-Biotin-THF) to expand the application of the DRMBs as an endpoint measurement method for a quick analysis of the activities of DNA repair enzymes (Figure 6A). The T at the loop region of the DRMB-THF2 was replaced with a biotin labeled T, which is used for the conjugation of the beacon to streptavidin beads. We also switched the CC-FAM at the 5′ end of the DRMB-THF2 beacon to the 3’ end of the new substrate, while placing a GG-Iowa Black^®^ Dark quencher at the 5′ end. With these modifications, after cleavage of the beacon and release of the quencher, the fluorescent signal will remain on the beads for signal measurement by flow cytometry. As expected, the DRMB-Biotin-THF was effectively cleaved by the purified APE1 enzyme (Figure 6B, blue line), with an activity profile similar to that of the DRMB-THF2 substrate (Figure 6B, red line).

To optimize the ratio of the beads and beacon, we mixed different volumes of fully cleaved DRMB-Biotin-THF with streptavidin beads (as described in the Materials and Methods) and measured the fluorescent signal by flow cytometry (Supplementary Figure 6). The uncut DRMB-Biotin-THF-conjugated beads were used as the negative control. Flow cytometry analysis revealed that 99.2% of the beads were fluorescently-labeled when using 1 pmol of cleaved DRMB-Biotin-THF, which was selected for all subsequent experiments. Further increases in the amount of DRMB-Biotin-THF did not show significant improvement (not shown). Using this beacon-bead ratio (4.5 × 10^5^ bead/1pmol beacon), we were able to differentiate the cleavage percentage of DRMB-Biotin-THF captured by streptavidin beads. We combined uncut DRMB-Biotin-THF with fully cut DRMB-Biotin-THF at different ratios and then the mixtures were captured by streptavidin beads and analyzed by flow cytometry. There is greater than a seven-fold change in the geo mean value of fluorescent signal on the fully cleaved DRMB-Biotin-THF compared to the uncut DRMB-Biotin-THF captured by streptavidin beads (Supplementary Figure 6). The geo mean values of fluorescent signals increased with the percentage of the cleaved DRMB-Biotin-THF in a linear fashion (R^2^ = 0.9958) (Figure 6C), and this linearity and dynamic range supports flow cytometry based quantifications for such DRMB platforms.

Next, we performed an APE1 activity assay using the newly developed on-bead cleavage of the DRMB-Biotin-THF followed by flow cytometry analysis. In this analysis, the DRMB-Biotin-THF was conjugated with streptavidin beads first, and then the beacon-bead conjugate was added to the beacon assay reactions with or without purified APE1 protein. After 2 hours of cleavage, the beads were washed and analyzed via flow cytometry. As shown in Figure 6D, the DRMB-Biotin-THF conjugated bead system successfully detected the activity of APE1, with the analysis indicating a 5.6-fold increase over that of the no APE1 control. We also performed the APE1 activity assay using the DRMB-Biotin-THF conjugated bead system with cell lysate from U2OS/APE1-KD or U2OS/SCR (control) cells (Figure 6E). As shown (Figure 6F), the analysis reveals a 4.4-fold decrease in APE1 activity in the lysate from the U2OS/APE1-KD cells (APE1 depleted by shRNA) relative to the U2OS/SCR (control) cells. These results indicate that the beacon conjugated-beads, combined with flow cytometry analysis, can be used as a new tool to measure the activities of DNA repair enzymes, using either purified proteins or cell/tissue lysates.

## DISCUSSION

A common phenotypic thread among many of the cellular and organismal alterations that arise in cancer, disease and aging is a defect in DNA repair capacity and a defect in the response to endogenous or environmental stressors [65–70]. Many of these cancer- or disease-specific DNA repair defects [25] can be detected using current “omics” technologies such as evaluating changes in mRNA expression or alterations in the DNA coding sequence. However, there are many defects that can only be detected from a direct analysis of protein function.

Because of the critical roles played by DNA repair genes/proteins in tumorigenesis and cancer therapy, we developed the DRMB platform to quantitatively measure the activity of select DNA repair proteins and DNA repair capacity of cells/tissues. Compared to the classical, acrylamide gel-based activity assay historically used in the analysis of many BER proteins [71], this fluorescent activity assay avoids the need to use gel electrophoresis, utilizes substrates that are stable for > 6 months (or longer pending proper storage) and is performed in a 96-well plate with over 300 data points per well that enable enzyme or repair kinetic analyses and allows the measurement of multiple assays simultaneously [15, 34]. Recognizing the potential significance of BER activity quantification for oncology, we aimed at improving the DRMB assay to a level that allowed the detection of small but potentially clinically relevant alterations in DNA repair capacities. Therefore, to further increase the signal-to-noise ratio, we modified the original beacon design by replacement of the 5′ guanosine linked to 6-FAM with a cytosine (Figure 1A), reducing the quenching effect of the guanosine base. This substrate modification increased the fluorescent signal greater than 3.5-fold in assays involving a THF lesion and nuclear lysates from LN428 cells (Figure 1B) or purified APE1 protein (Supplementary Figure 4). The DNA repair activity of multiple lesion-specific DNA glycosylases, such as MPG, SMUG1, UNG, NTH1, NEIL1 and OGG1 could be readily detected in lysates from human tumor cells (LN428, U2OS and K562 cell lines) and normal cells (PBMCs).

For the DRMBs described here, the design mimics the BER pathway, whereby the glycosylase removes the base lesion, leaving the DNA backbone intact. Subsequently, beacon DNA cleavage and signal amplification are dependent on APE1, which releases the fluorophore from the quencher. The need for APE1 activity in the context of the BER-based DRMB assay was confirmed using purified mono-functional MPG and APE1 proteins and an Hx molecular beacon (DRMB-Hx) substrate. Mono-functional glycosylases excise the substrate base, but leave an intact AP site as a substrate for APE1. Fluorescent signal was thus only generated when both MPG and APE1 proteins were present. For the BER-based DRMB assay using cell lysates, APE1 is also a critical endonuclease, as depletion (knockdown) of APE1 in LN428 and U2OS cells strongly reduced AP site cleavage activity in the DRMB-THF2 assay. Consistent with the MPG results, loss of APE1 (APE1-KD) strongly reduced the measurable activity of UNG, another mono-functional DNA glycosylase, in the DRMB-dU/A assay. Thus, the signal of the DRMB-dU/A assay is dependent on APE1 to cleave the AP site generated by UNG. Together, these analyses show the APE1-dependence in detecting BER glycosylase activities using the DRMB assay. The inclusion of samples with added purified APE1 in the DRMB platform could therefore reveal variations in upstream activities in samples in which APE1 is rate-limiting.

In earlier work, it was shown that similar repair beacons may show utility to evaluate repair capacity in live cells [36]. In our hands, transfection of the DRMB-Con2 and DRMB-THF2 probes into cells revealed an increase in signal within 30 minutes, peaking around 2 hours (Supplementary Figure 5). However, this appeared to be the result of non-specific nucleases as the same signal intensity was observed for both the control and APE1 substrates. Further effort will be needed to optimize these DRMB probes for *in vivo* (in cell) activity analysis.

The bi-functional glycosylase NTH1 is considered to be the major glycosylase for the removal of the mutagenic and cytotoxic DNA lesion Tg, with other glycosylases functioning as backup enzymes for the repair of this lesion, albeit at a slower rate [72, 73] (Table 3). It is surprising therefore that the activity of NTH1 was strongly decreased in the DRMB-Tg assay after APE1-KD (Figure 3F). Bi-functional DNA glycosylases (Figure 3D) have the capacity to cleave the AP site after base excision via a β- or β,δ-elimination reaction using the ε-NH2 of a lysine or the N-terminal amino acid residue proline as the active site nucleophile without the help of APE1 [38]. However, some bi-functional DNA glycosylases such as OGG1 process AP sites poorly and intact AP sites are the major product after removal of the 8-oxoguanine lesion [74]. When APE1 is in excess, even an inactivated APE1 at a higher concentration, there is an approximate 5-fold increase in the specific activity of OGG1 [74]. Kinetic studies have shown that OGG1 tends to bind tightly to its AP site product following base excision, thus reducing turnover. APE1 promotes dissociation of OGG1 from the AP site, thus enhancing turnover [74]. The same mechanism was also reported for NTH1, whereby APE1 increased the activity of NTH1 by enhancing turnover [75]. Our cell lysate results further support these findings as depletion of APE1 resulted in reduced strand cleavage following Tg lesion removal by NTH1 in the DRMB-Tg assay (Figure 3F), indicating that both mono-functional and bi-functional glycosylase activity and measured BER efficiency is influenced by APE1 presence.

Single amino acid substitution mutants can be identified by current DNA and RNA sequencing methodologies but these cannot define a change in function unless they occur in a defined active site location. Thus, enzyme activity analysis using our DRMB platform is a powerful tool in this regard. Due to the essential role of APE1 in the BER pathway, we evaluated the activities of ten purified APE1 proteins using the DRMB-THF2 assay. The results revealed that the activity of APE1 was impacted uniquely by the different single amino acid substitutions (Figure 4). The strategic E96A mutation abolished the activity of APE1, consistent with prior work demonstrating that mutation of the likely metal-coordinating residue results in a 500- to 600-fold decrease in enzymatic activity as compared to the wild type protein [61, 76]. As we observed herein, prior work reported that a G241R substitution slightly enhances the endonuclease activity of APE1 [77]. However, regarding the polymorphic variant D148E, as well as the variants Q51H and I64V, previous biochemical studies did not reveal a repair defect in multiple assays [77], suggesting that the slight decrease in activity measured via the DRMB-THF2 assay may stem from the non-conventional reaction conditions (i.e., lack of Mg^2+^ ions) or the assay's design, sensitivity or real-time resolution. As for the tumor-associated variant R237C, independent studies have described a defect in AP-site binding and processing [78], specifically in the context of pre-assembled protein-DNA complexes, as well as an inability to effectively complement APE1-deficiency in terms of cell growth and genotoxin resistance [53, 79]. Our observation that R237C has enhanced activity in the DRMB assay likely reflects the altered interaction of R237C with DNA, which is apparently affected in different ways by the specific substrate design, reaction parameters or end-point analysis. Further work is required to clarify these apparently contradictory findings, perhaps focusing on the effects of magnesium concentrations on the real-time activities of select APE1 protein variants.

An advantage with our DRMB assay is the ability to detect possible changes in the activities of DNA repair enzymes in tumor cells as compared to normal cells, such as PBMCs. The ability to evaluate PBMCs as we show suggests that we can evaluate changes in DNA repair capacity in future population studies. Notably, prior studies have indicated that altered BER capacity can lead to a higher incidence of cancer, such as the elevated colon cancer risk for MUTYH mutant carriers [80]. Further, the analysis of 300 head and neck cancer (HNC) cases and 300 matched healthy controls showed that *APE1* mRNA expression was positively correlated with tumor size, clinical stage and positive lymph node metastasis [54]. In addition, there is an apparent upregulation in the expression of the *UNG* gene in some leukemias, as shown by gene expression profiling analysis of 170 acute myeloid leukemia (AML) patients [81]. Our DRMB assays show very precise and quantitative analysis of base lesion repair capacity, indicating that the assay may be of value when evaluating the molecular profile of cancer patient samples.

After demonstrating the high sensitivity of this DRMB assay on the measurement of the activities of several BER enzymes, we modified the beacon oligo and extended the application of this technique for use in flow cytometry, a routine lab-based technology and one that is now utilized as a frontline component of modern blood cancer diagnosis [82]. The newly designed DRMB-Biotin-THF assay for flow cytometry showed similar sensitivity as the DRMB-THF2 assay, and was demonstrated to work with both purified protein and cell lysates. Additional optimization to allow multiplexing will further improve the sensitivity and utility of this novel approach.

In summary, we report here the development of an activity assay for DNA repair enzymes, which can be readily performed using standard lab equipment. The assay is sensitive enough to detect the activity change of the enzyme APE1 harboring single amino acid substitutions and variations in expression of DNA glycosylases or APE1 in cell lysates. Given the reported variation in BER protein expression and gene mutations/deletions across all cancers (Supplementary Figure 7) [83, 84], this approach will be a valuable tool towards a complete analysis of DNA repair capacity in tissues and cells across cancer and disease cohorts. We suggest that the application of this assay may be considered as a diagnostic tool to identify disease risk, to develop precise treatments, as a surveillance tool to monitor disease status as well as functionally characterizing tumor cells and tissue at the molecular level to aid personalized therapeutic strategies.

## MATERIALS AND METHODS

### Cell lines

LN428 glioblastoma cells were cultured in alpha MEM supplemented with 10% heat inactivated FBS, L-glutamine, antibiotic/antimytotic and gentamycin, as we have described [85, 86]. U2OS cells were obtained from ATCC and were cultured in DMEM with 10% heat inactivated FBS, L-glutamine and antibiotic/antimytotic. The K562 human chronic myelogenous leukemia cell line was obtained from ATCC and was cultured in RPMI 1640 medium with 10% fetal bovine serum as described previously [87]. All cells were cultured in a humidified incubator at 37°C, 5% CO_2_.

### APE1-KD and SCR control cell lines

APE1-KD cells were developed by transduction of LN428 or U2OS cells with lentivirus expressing APE1-specific shRNA, essentially as described [85, 86]. Briefly, lentiviral vectors expressing shRNA specific to APE1 (NM_080649.1-1305s1c1, CAGAGAAATCTGCATTCTATT) or the corresponding scrambled shRNA (SCR) control were obtained from Sigma and prepared by the MCI Gene Expression, Engineering and Discovery (GEED) Facility. Lentiviral particles were generated by co-transfection of 4 plasmids [SCR or APE1-specific shRNA plasmid (pLK0-Puro) with pMD2.g (VSVG), pVSV-REV and pMDLg/pRRE] into 293-FT cells using TransIT-X2^®^ Dynamic Delivery System (Mirus Bio LLC). For transduction, LN428 or U2OS cells (1 × 10^5^) were seeded into a 6-well plate 24 hrs before transduction, and then transduced with the shRNA lentiviruses at 32°C overnight. After incubated at 37°C for an additional 24 hrs, cells were selected in growth media containing puromycin (1.0 μg/ml) for two weeks. APE1-KD was validated by qRT-PCR using an Applied Biosystems StepOnePlus system via the ΔΔCT method. The Taqman probe for APE1 (Hs00172396_m1) was purchased from ThermoFisher Scientific. cDNA was prepared using the Taqman Gene Expression Cells-to-CT kit (ThermoFisher Scientific) according to the manufacturer's instructions. Statistical analysis was accomplished using GraphPad Prism by one-way ANOVA with Tukey as a *post hoc* test (multiple comparisons).

Immunoblotting analysis was performed by separating nuclear protein or whole cell lysate by 4–12% SDS-PAGE and electro-transferred to a 0.45 mm nitrocellulose membrane (Trans-Blot, Bio-Rad). Antigens were detected using standard protocols. The primary antibody, anti-APE1 (Abcam, # ab137708), was used at a 1:1000 dilution in TBST / 5% milk and the membrane was incubated overnight (4°C). The HRP conjugated secondary antibody (GAM-HRP or GAR-HRP, Bio-Rad) was diluted 1: 5000 in TBST / 5% milk. Images were achieved using a ChemiDoc™ XRS+ System (BioRad).

### Preparation of cell pellets and lysates

Cells (LN428, U2OS) were trypsinized and washed twice with PBS. K562 cells (suspension cultures) were pelleted by centrifugation (200xg) and washed twice with PBS. After the removal of PBS, cells were pelleted by centrifugation (200xg) and stored at −80°C before lysate preparation. Peripheral blood mononuclear cells (PBMCs) were obtained from the University of South Alabama Mitchell Cancer Institute (MCI) Biobank. Informed consent was obtained from a healthy, non-smoking male volunteer and then blood was obtained. All methods to obtain and isolate these PBMCs were obtained in accordance with the relevant guidelines and regulations of the University of South Alabama (USA) Institutional Review Board (IRB). All experimental protocols for the isolation and preparation of the PBMCs were approved by the USA/IRB committee – as defined in the USA/MCI protocol # 03-092. Once obtained, the blood was transferred into BD vacutainer^®^ CPT mononuclear preparation tubes (BD) containing the anti-coagulant sodium heparin and blood separation media composed of a thixotropic gel and FICOLL™ Hypaque™ solution. Samples were centrifuged at RT at 1500 relative centrifugal force (rcf) for 15 min using a swinging bucket rotor (5810R, Eppendorf, Hauppauge, NY). After centrifugation, the lymphocytes and mononuclear cells were visible as a white layer that was collected using a Pasteur pipette. PBMCs were recovered, washed twice with 10 ml PBS, pelleted by centrifugation (200xg) and stored at −80°C before lysate preparation. Nuclear protein extracts were prepared using the NucBuster Protein Extraction Kit (Calbiochem, # 71183). The nuclear preparations were dialyzed and diluted to 2 μg/μl as previously described [15, 34]. Whole cell lysates were prepared by ultrasonication. Each cell pellet was re-suspended in 250 μl of molecular beacon reaction buffer with protease inhibitors as per the manufacturer's instructions (Pierce, #539131) [15, 33, 34]. Then, cell suspensions were sonicated once for 5 seconds with 50% amplitude on ice followed by a centrifugation at the highest speed for 15 min at 4°C to remove debris. Protein concentration was determined using Bio-Rad protein assay reagents according to the manufacturer's instructions.

### Recombinant protein purification

For the expression and purification of His-APE and His-MPG described in Supplementary Figure 2 and analyzed in Figure 3A, human APE1 (full length) and MPG(Δ26) cDNA were cloned into the pENTR/D-TOPO plasmid to create the pENTR-APE1 and pENTR-MPG(Δ26) vectors, as per standard Topo-cloning methodology, each without a start codon. Each ORF was then transferred to the pDEST17 expression vector by the LR reaction using the Gateway LR Clonase II Enzyme Mix (Thermo Fisher Scientific) to generate pDEST17-APE1 and pDEST17-MPG(Δ26), allowing expression of the His-Ape1 and His-MPG(Δ26) proteins in *E. coli*, as we have described [17]. His-APE1 and His-MPG(Δ26) were expressed in BL21(DE3) cells following induction by isopropyl β-d-thiogalactopyranoside (IPTG) at 37°C, overnight. His-APE1 and His-MPG(Δ26) proteins were purified using the HisTalon kit (Cat# 635654, Clontech). After purification, His-APE1 protein was dialyzed into storage buffer A: [50 mM NaCl, 10 mM Tris-HCl (pH 8.0), 0.05 mM EDTA, 1 mM dithiothreitol, 200 μg/ml BSA and 50% glycerol] and kept at −80°C until assayed. His-MPG(Δ26) protein was dialyzed into storage buffer B: [100 mM KCl, 10 mM Tris-HCL (pH7.4), 0.1 mM EDTA, 1 mM dithiothreitol, 0.5% Tween 20, 0.5% NP-40 and 50% glycerol] and kept at −80°C until assayed.

All APE1 proteins described in Figure 4 were expressed in bacteria following induction by IPTG at 37°C and purified as described previously [88]. Briefly, fractions eluting from the cation exchange S10-column containing wild-type or variant APE1 proteins were concentrated using a centricon-10 filtration device (Amicon, Bedford, MA) and further separated on a gel-filtration column (Bio-Silect SEC125–5; Bio-Rad) in 50 mM HEPES, pH 7.5, 50 mM KCl and 5% glycerol. Protein concentration was determined using Bio-Rad protein assay reagents according to the manufacturer's instructions and further analyzed by immunoblot using an anti-APE1 antibody (Abcam, # ab137708). Protein quantification was achieved by Image J-FIJI [89].

### Beacon preparation

All of the oligonucleotides used to create the molecular beacons were ordered from Integrated DNA Technologies and purified by HPLC. All oligonucleotides were dissolved, annealed and diluted as previously described [15, 34]. Once annealed and diluted, the beacons were assessed using a melt curve experiment to validate similar secondary structure and to determine optimal melting temperatures to be used in future normalization steps [34].

### DNA Repair Molecular Beacon (DRMB) assay

The DRMB assay was run using a StepOnePlus qRT-PCR machine from Applied Biosystems at 37°C. Fluorescence was measured for three technical replicates every 20 seconds for 1 or 2 hours, before increasing the temperature for ‘melt curve’ normalization and acquisition of fluorescence values from unwound beacons, as previously described [34]. The ‘melt curve’ fluorescence values from each well were used to normalize the absolute fluorescence values collected as per previously published methods [34]. Briefly, the ‘melt curve’ analysis is run at the completion of the enzymatic analysis (60 or 120 minutes). The reactions are heated in 5-minute step-wise reactions, at increasing temperatures, ranging from 60-90°C to determine the maximum fluorescence, Fl(T_max_), for each well of each beacon. This allows normalization of the data to the maximal fluorescence values (that correlate with beacon input). These normalized data represent % free FAM (= % BER incised beacon). The normalized fluorescence values are plotted and are the mean of two or three independent experiments with error bars denoting range or standard error of the mean (SEM), respectively. For the DRMB assay with Mg^2+^, the reaction buffer was modified to include 5 mM MgCl_2_.

### Flow cytometry analysis of cleaved DRMB-Biotin-THF captured by streptavidin microsphere beads

DRMB-Biotin-THF (1 pmole) was cleaved by purified WT His-APE1 protein in a 25 μl reaction as previously described [34] (group A). Uncut DRMB-Biotin-THF (1 pmole) was used as the negative control (group B). We mixed group A and B at 100%A:0%B; 75%A:25%B; 25%A:75%B; or 0%A:100%B to mimic a range from fully cleaved to total un-cleaved beacon. Streptavidin Microsphere Beads (5 μl = 4.5×10^5^ beads) (Polysciences 6.0 μm, cat#24158-1) were washed three times with molecular beacon reaction buffer containing protease inhibitors [15, 33, 34] and re-suspended in 25 μl/well of molecular beacon reaction buffer. Beads were then mixed with each group as described above. The mixed suspensions were shaken at room temperature for 30 minutes. Next, the conjugated bead-beacon was washed and re-suspended in PBS and analyzed by Flow cytometry at the MCI Flow Cytometry Core Laboratory.

### Flow cytometry analysis of on-bead cleavage of the DRMB-Biotin-THF conjugated to streptavidin microsphere beads by purified APE1 protein

For each on-bead molecular beacon assay, 5 μl streptavidin Microsphere Beads (6.0μm, cat# 24158-1, Polysciences) were washed three times with molecular beacon reaction buffer containing protease inhibitors [15, 33, 34] and re-suspended in 25 μl/well of molecular beacon reaction buffer. Beads were then mixed with beacon (5 μl, 200nM) and shaken at RT for 30 minutes. The conjugated bead-beacon was then washed twice with molecular beacon reaction buffer and re-suspended in 20 μl molecular beacon reaction buffer. The molecular beacon assay was initiated by adding 5 μl APE1 protein to the 20 μl conjugated bead-beacon suspension and kept at 37°C for 2 hours. After the completion of the reaction, the mixture was washed and re-suspended in PBS and analyzed on a BD Biosciences Canto II cytometer at the MCI Flow Cytometry Core Laboratory. Events (10,000) were collected and fluorescent signal measured in the Blue E channel (15mW 488nm excitation, 530/30nm emission). Data is represented as MFI (mean fluorescence intensity-geometric mean).

### Flow cytometry analysis of on-bead cleavage of the DRMB-Biotin-THF conjugated to streptavidin microsphere beads by cell lysate from U2OS/APE1-KD or U2OS/SCR cells

For the on-bead molecular beacon assay, 50 μl streptavidin microsphere beads (6.0μm, cat# 24158-1, Polysciences) were washed once with molecular beacon reaction buffer containing protease inhibitors [15, 33, 34] and re-suspended in 150 μl DRMB-Biotin (200nM) and kept at RT for 10 minutes. The conjugated bead-beacon was then washed three times with molecular beacon reaction buffer and re-suspended in 50 μl molecular beacon reaction buffer. Whole cell lysates were prepared by ultrasonication as described above and protein concentration was determined by the Quick Start™ Bradford Protein Assay (cat#5000201, Bio-Rad). For each DRMB assay, 5 μl conjugated bead-beacon suspension was added to the 30 μl cell lysate and kept at 37°C for 2 minutes. Then, the mixture was re-suspended in 300 μl PBS and analyzed immediately on a BD Biosciences Canto II cytometer at the MCI Flow Cytometry Core Laboratory. Events (10,000) were collected and fluorescent signal measured in the Blue E channel (15mW 488nm excitation, 530/30nm emission). Data is represented as MFI (mean fluorescence intensity-geometric mean).

### Data analysis and statistics

Data were collected and fluorescence values normalized (FL_N_) for each well as described above. FL_N_ values of 3 intra-experimental replicates (technical replicates) were averaged and mean values of the experiments used to assess and compare the experimental conditions. Multiple parameters were calculated to compare repair activities: i) Area Under the Curve (AUC) values were calculated using the GraphPad Prism software package and served for overall repair difference comparisons; ii) incision rates and iii) maximal substrate repair (maximal incised beacon fraction) were deferred from the FAM-labelled nucleotide dissociation rate constants (k) and maximal dissociation values (Y_max_, FL_N_ at indefinite time point x) from exponential curve fits generated by GraphPad Prism with:

*Y* = *Y_max_* * (1 − *e*^−*kx*^).

Best fit values for k or Y_max_ were tested using extra sum-of squares F test with p<0.05 to assess whether they differed. Differences in mean AUC were assessed using one way ANOVA (GraphPad Prism software). AUC, k, Y_max_ and statistical values are summarized in Supplementary Tables 1 and 2. Error bars or bands on the graphs in the figures indicate the range or the standard error of the mean (SEM) on the independent experimental values (biological replicates) as indicated.

## SUPPLEMENTARY MATERIALS FIGURES AND TABLES




