# Supplementary material for: DNA Repair Molecular Beacon assay: a platform for real-time functional analysis of cellular DNA repair capacity
Source: Oncotarget. 2018 Aug 3;9(60):31719–43. doi: 10.18632/oncotarget.25859 (PMC6114979; doi:10.18632/oncotarget.25859)
Supplement: Supplementary file 1 [file oncotarget-09-31719-s001.pdf]

DNA Repair Molecular Beacon assay: a platform for real-time functional analysis of cellular DNA repair capacity

SUPPLEMENTARY MATERIALS

In vivo DNA Repair Molecular Beacon (DRMB) assay in living cells

The method for the *in vivo* DRMB assay was modified from the protocol described by Maksimenko et al. [1]. The U2OS/SCR cells and U2OS/APE1-KD cells were seeded in 4-well chamber slides (20,000 cells/well) and cultured overnight (37˚C). Before transfection, the culture medium was replaced with 1 ml fresh growth medium. Two hundred picomoles of each DRMB (DRMB-Con2 or DRMB-THF2) and 2 μl of TransIT-X2 (Cat# MIR6003 Mirus Bio™) were diluted separately in 50 μl of transfection solution (100mM NaCl, 10mM Hepes, pH 7.5) and then were mixed together. After 15 min incubation at room temperature, TransIT-X2-DRMB complexes were added drop-by-drop to the cells. From 30 minutes to 2 hrs, the slides were analyzed by confocal fluorescence microscopy (Nikon Ti-E with A1rsi) for live-cell image analysis in the USA MCI Cellular and Biomolecular Imaging Facility. Images are representative of three independent experiments at the 0 hr and 2 hr time-point.

REFERENCES

1. Maksimenko A, Ishchenko AA, Sanz G, Laval J, Elder RH, Saparbaev MK. A molecular beacon assay for measuring base excision repair activities. Biochem Biophys Res Commun. 2004; 319:240–6. https://doi.org/10.1016/j.bbrc.2004.04.179.
2. Chandran UR, Luthra S, Santana-Santos L, Mao P, Kim SH, Minata M, Li J, Benos PV, DeWang M, Hu B, Cheng SY, Nakano I, Sobol RW. Gene expression profiling distinguishes proneural glioma stem cells from mesenchymal glioma stem cells. Genom Data. 2015; 5:333–6. https://doi.org/10.1016/j.gdata.2015.07.007.
3. Mao P, Joshi K, Li J, Kim SH, Li P, Santana-Santos L, Luthra S, Chandran UR, Benos PV, Smith L, Wang M, Hu B, Cheng SY, et al. Mesenchymal glioma stem cells are maintained by activated glycolytic metabolism involving aldehyde dehydrogenase 1A3. Proc Natl Acad Sci U S A. 2013; 110:8644–9. https://doi.org/10.1073/pnas.1221478110.
4. Gao J, Aksoy BA, Dogrusoz U, Dresdner G, Gross B, Sumer SO, Sun Y, Jacobsen A, Sinha R, Larsson E, Cerami E, Sander C, Schultz N. Integrative analysis of complex cancer genomics and clinical profiles using the cBioPortal. Sci Signal. 2013; 6:pl1. https://doi.org/10.1126/scisignal.2004088.
5. Cerami E, Gao J, Dogrusoz U, Gross BE, Sumer SO, Aksoy BA, Jacobsen A, Byrne CJ, Heuer ML, Larsson E, Antipin Y, Reva B, Goldberg AP, et al. The cBio cancer genomics portal: an open platform for exploring multidimensional cancer genomics data. Cancer Discov. 2012; 2:401–4. https://doi.org/10.1158/2159-8290.CD-12-0095.

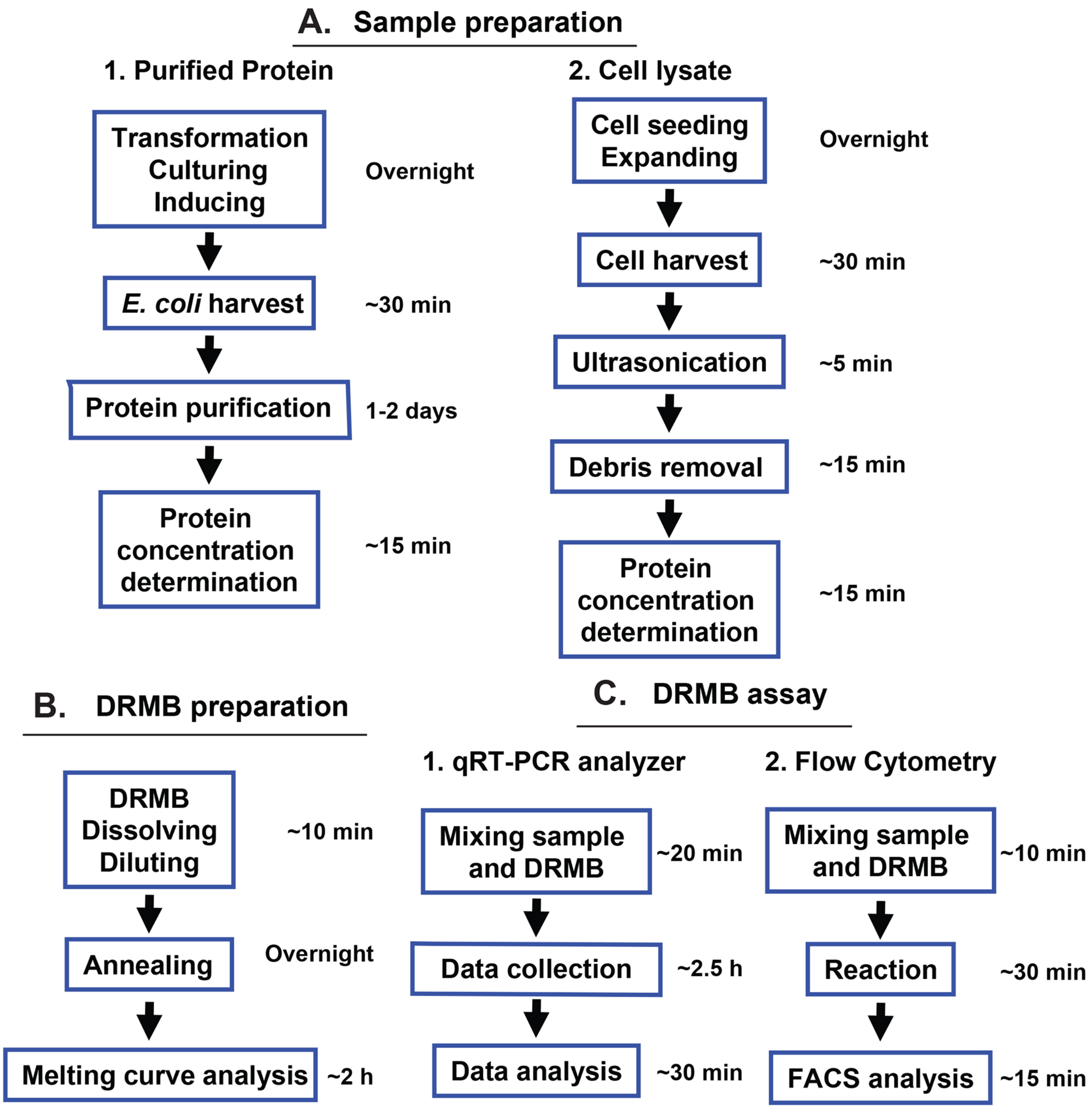

Supplementary Figure 1: Time frame for performing the DRMB assay. **(A)** Sample preparation for DRMB assay: Proteins are purified from transformed *E. Coli* or cell lysates are prepared from cultured cells using an ultrasonic homogenizer. **(B)** Substrate preparation for DRMB assay: DRMB oligos (40μM) are diluted with beacon reaction buffer to the working concentration (200nM) and then annealed overnight. Each DRMB oligo is initially validated for fluorescence using a melt curve experiment. **(C)** Performing a DRMB assay: Using a qRT-PCR instrument, each sample and DRMB are mixed in the well of a 96-well plate and the fluorescence intensity of each well is recorded every 20 seconds for the length of the assay (60-120 minutes). Using Flow Cytometry, DRMB substrates are captured by streptavidin microsphere beads and mixed with each sample in a 5 ml round bottom tube. The tubes are kept at either room temperature or 37°C for a period of time for the excision of the DRMB. Then, the DRMB conjugated beads are washed and the fluorescence intensities of the beads are analyzed using a Flow Cytometer.

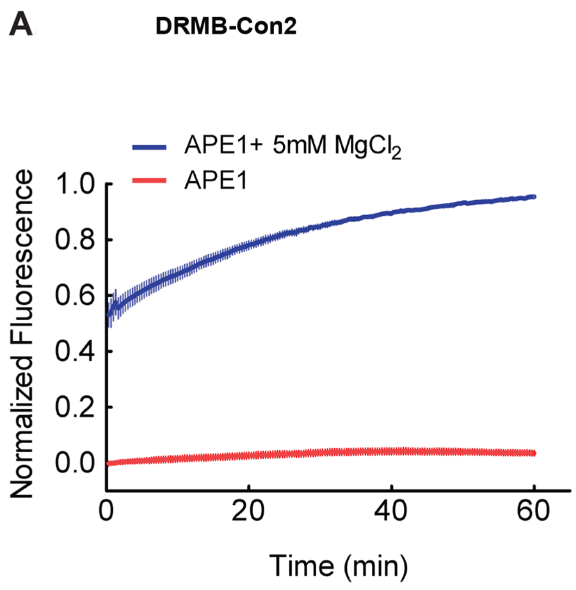

Supplementary Figure 2: DRMB assay with purified APE1 protein supplemented with Mg^{2+}. (A) DRMB-Con2 assay using purified recombinant APE1 protein with or without 5 mM $MgCl_2$. **(B)** DRMB-THF2 assay using purified recombinant APE1 protein with or without 5 mM $MgCl_2$.

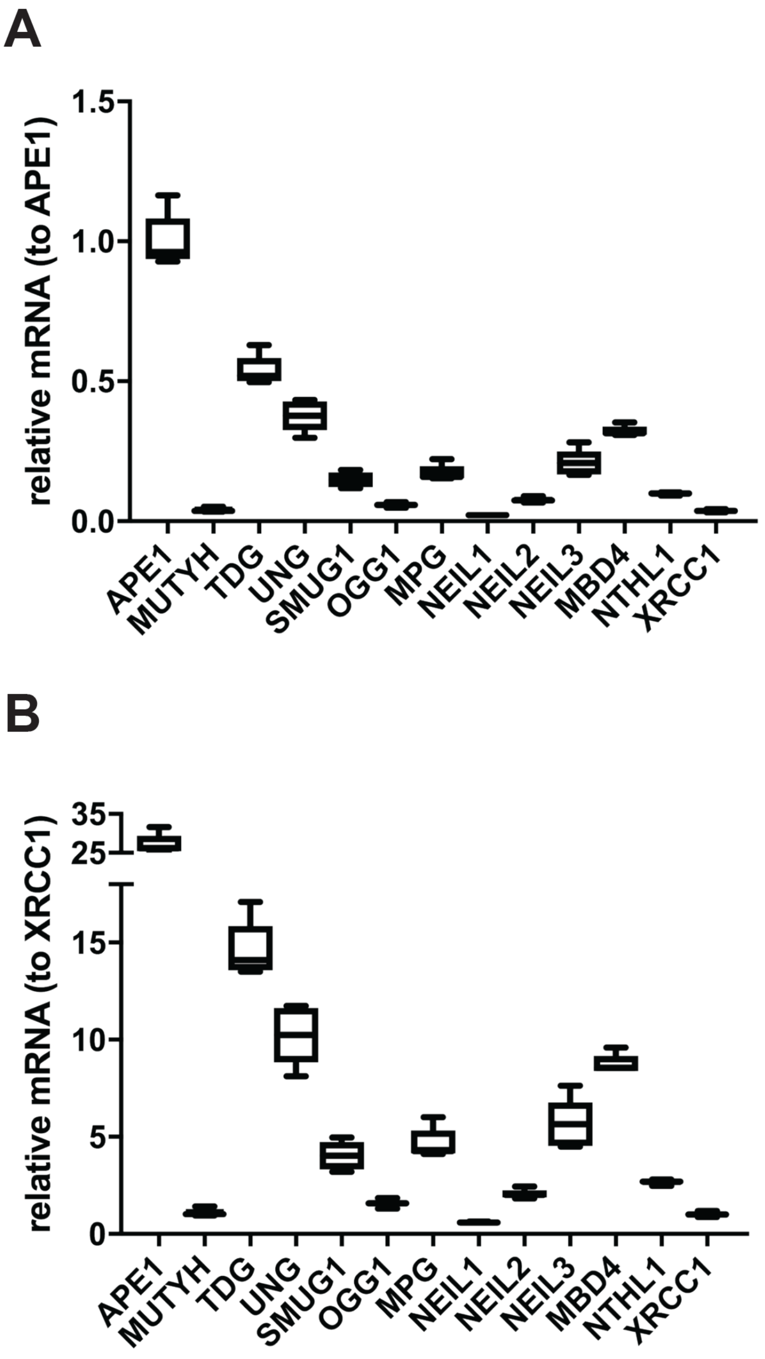

Supplementary Figure 3: Relative mRNA level of DNA glycosylases in LN428 cells. The mRNA profiles of LN428 mRNA samples from four different cultures were extracted from microarray analysis data using the Human U219 Array Strip and the Affymetrix GeneAtlas system, as we have described previously [2, 3]. Shown are whisker plots of relative mRNA levels normalized to APE1 **(A)** or XRCC1 **(B)**.

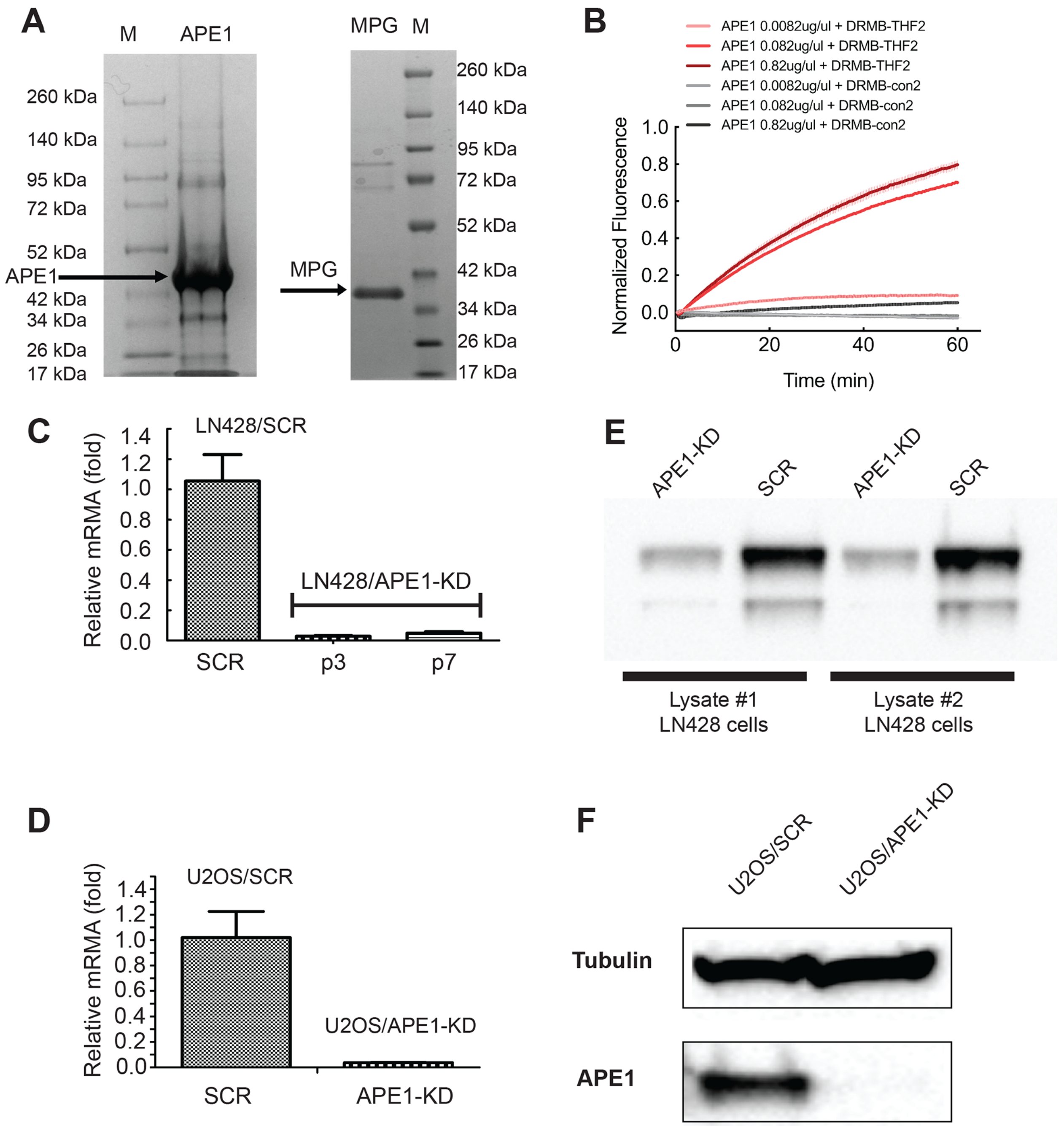

Supplementary Figure 4: Expression and purification of recombinant human APE1 and MPG in *E. coli* and the characterization of APE1 mRNA / protein in stable APE1-deficient human cell lines. (A) Human APE1 protein (left) was expressed in BL21 *E. coli* cells and purified by a TALON Metal Affinity Resin followed by a Heparin column. Human MPG protein (right) was expressed in BL21 *E. coli* cells and purified by a TALON Metal Affinity Resin. **(B)** DRMB-THF2 assay using purified recombinant APE1 protein. **(C-D)** APE1 mRNA levels, as determined by qRT-PCR, in LN428 cells after 3 and 7 passages (C) and in U2OS cells 10 passages after transduction (D). APE1 immunoblots comparing LN428/SCR (scrambled controls) to LN428/APE1-KD cells **(E)** and U2OS/SCR to U2OS/APE1-KD cells **(F)** and showing depletion of APE1 protein mediated by shRNA.

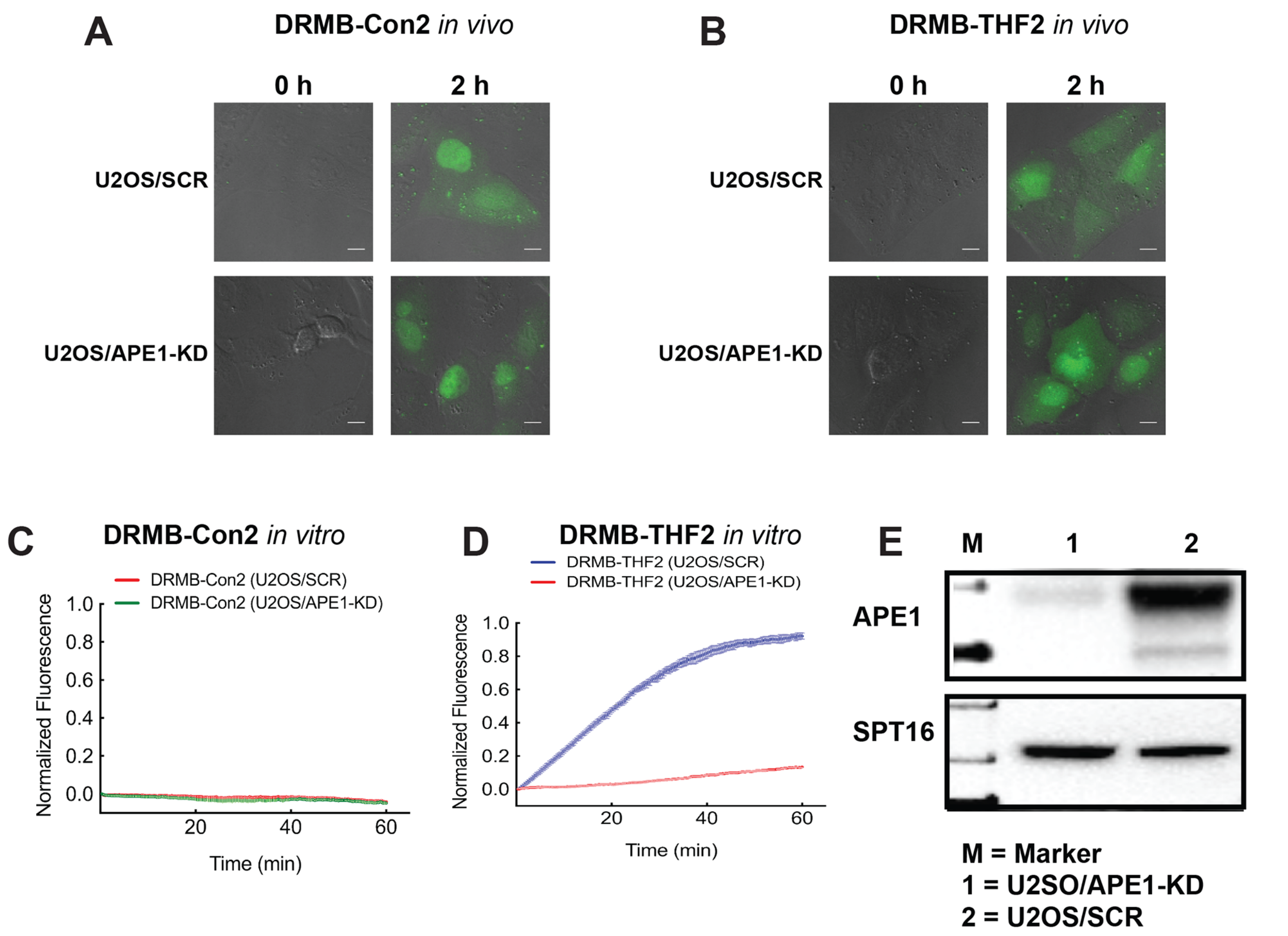

Supplementary Figure 5: *In vivo* DRMB assay in APE1 depleted cells. (A) DRMB-Con2 was transfected into U2OS/SCR or U2OS/APE1-KD cells and the images were taken at 0h and 2h after transfection. **(B)** DRMB-THF2 was transfected into U2OS/SCR or U2OS/APE1-KD cells and the images were taken at 0h and 2h after transfection. **(C)** DRMB-Con2 *in vitro* assay using cell lysate from U2OS/SCR or U2OS/APE1-KD cells. **(D)** DRMB-THF2 *in vitro* assay using cell lysate from U2OS/SCR or U2OS/APE1-KD cells. **(E)** Immunoblots showing depletion of APE1 protein mediated by shRNA in U2OS/APE1-KD cells compared to U2OS/SCR cells. SPT16 blot used as a non-targeted protein loading control.

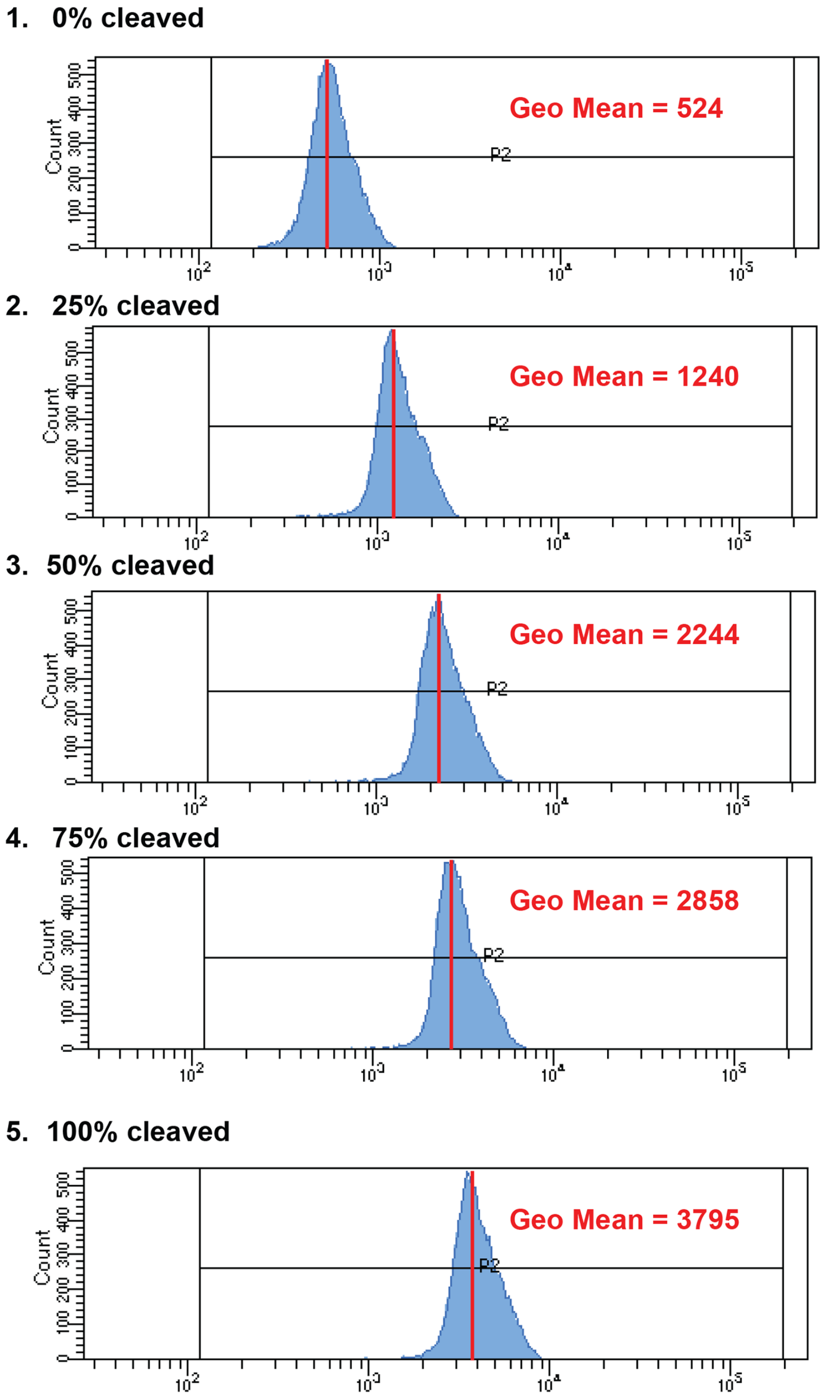

Supplementary Figure 6: Analysis of microbead-captured cleaved DRMB-Biotin-THF followed by flow cytometric analysis. DRMB-Biotin-THF cleaved by APE1 protein after 60min as shown in Figure 6B was defined as 100% cleaved. Cleaved and un-cleaved DRMB-Biotin-THF were mixed at different ratios, as indicated. Mixtures were captured by streptavidin beads and subsequently analyzed by flow cytometry. The ratio of cleaved and un-cleaved DRMB-Biotin-THF were then differentiated by the geometric mean fluorescence intensity value of the beads (Geo mean).

A

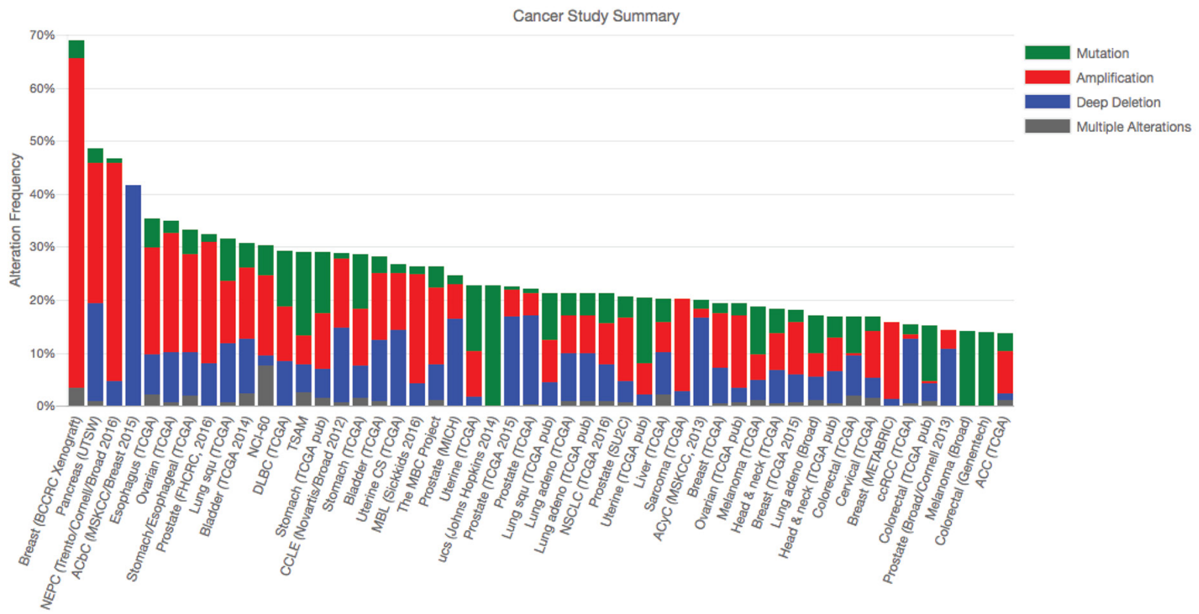

B

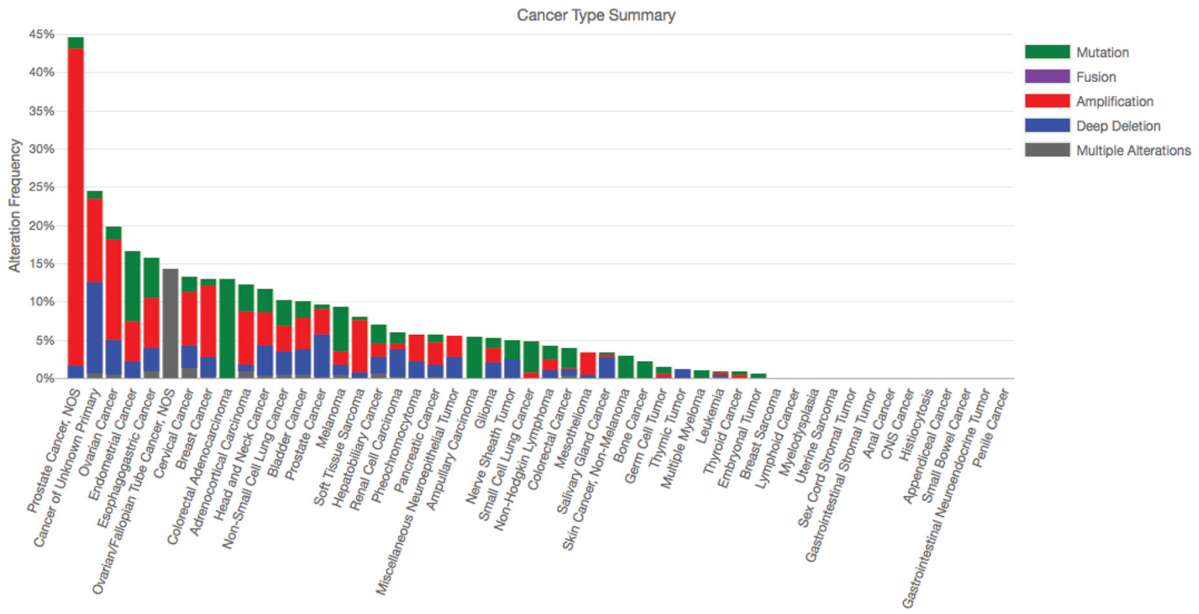

Supplementary Figure 7: Integrated genomic analysis using the cBio portal to evaluate BER genes. An analysis [4, 5] of mutations, amplifications and deep deletions for OGG1, TDG, UNG, SMUG1, NTHL1, NEIL1, NEIL2, NEIL3, MPG, MBD4, MUTYH and APE1 were ranked by **(A)** cancer study and **(B)** cancer type.

Supplementary Table 1: Statistical p values

Figure		*p* values k	AUC	Ymax	n
1B	DRMB-THF vs DRMB-THF2	<0.001	ns	<0.001	2
2A	DRMB-THF/C vs DRMB-THF/T	<0.001	ns	<0.001	2
	DRMB-THF/C vs DRMB-THF/G	<0.001	ns	<0.001	2
	DRMB-THF/C vs DRMB-THF/A	<0.001	ns	<0.001	2
	DRMB-THF/G vs DRMB-THF/A	ns	ns	ns	2
	DRMB-THF/A vs DRMB-THF/T	0.0109	ns	ns	2
	DRMB-THF/G vs DRMB-THF/T	ns	ns	ns	2
2B	DRMB-8oxoG/C vs DRMB-8oxoG/A	<0.001	ns	<0.001	2
	DRMB-8oxoG/C vs DRMB-C/8oxoG	<0.001	ns	<0.001	2
	DRMB-8oxoG/C vs DRMB-A/8oxoG	<0.001	0.0142	<0.001	2
	DRMB-8oxoG/A vs DRMB-C/8oxoG	0.0453	ns	<0.001	2
	DRMB-8oxoG/A vs DRMB-A/8oxoG	0.3695	0.0398	<0.001	2
	DRMB-C/8oxoG vs DRMB-A/8oxoG	0.5063	ns	<0.001	2
3B	THF2 APE1-KD vs THF2 SCR (LN428)	*<0.0001*	0.0022	nd	3
3C	dU/A APE1-KD vs THF2 SCR (LN428)	*<0.0001*	0.0006	nd	3
3E	THF2 APE1-KD vs THF2 SCR (U2OS)	*<0.0001*	<0.0001	nd	3
3F	Tg APE1-KD vs Tg SCR (U2OS)	*<0.0001*	<0.0001	nd	3
4B	WT vs Q51H	<0.0001	0.0013	nd	3
	WT vs I64V	<0.0001	0.0152	nd	3
	WT vs E96A	<0.0001	<0.0001	nd	3
	WT vs P112L	<0.0001	0.181	nd	3
	WT vs D148E	<0.0001	0.0013	nd	3
	WT vs R237C	<0.0001	<0.0001	nd	3
	WT vs G241R	<0.0001	<0.0001	nd	3
	WT vs P311S	ns	ns	nd	3
	WT vs A317V	<0.0001	<0.0001	nd	3

The table summarizes the statistical test values corresponding to the individual Figures as indicated. Experimental conditions and beacons comparisons are indicated in the left column. DRMB assay data were fitted using the equation listed in the Supplementary Text. The extra sum-of squares F test with $p<0.05$ was used to compare the fits and test for significant differences for k or Y_{max} that were derived from the fits and are listed in Supplementary Table 2. Values based on linear fits are in italics. One-way ANOVA was used to test the differences in the mean AUC (area under the curve) values of the n (number) independent experiments/graphs.

Supplementary Table 2: List of excision parameters as derived from the DRMB-assay. Multiple parameters were calculated using the GraphPrism software package to compare excision activities: i) Area under the curve (AUC) values, ii) incision rates (k) and iii) maximal repair (Y_{max}). K and Y_{max} values were derived from "one-phase association" curve fits with: $Y = Y_{max} * (1 - e^{-kx})$. Standard errors (SE) and 95% confidence intervals (CI) are listed for each tested beacon. Best fits were tested and linear fits were applied when exponential fits were ambiguous or inferior. Linear fit derived values are in italics.

See Supplementary File 1
